# New global indicator for workers’ health: mortality rate from diseases attributable to selected occupational risk factors

**DOI:** 10.2471/BLT.23.289703

**Published:** 2023-05-01

**Authors:** Frank Pega, Rola Al-Emam, Bochen Cao, Cynthia W Davis, Sally J Edwards, Diana Gagliardi, Anaclaudia Gastal Fassa, Mohd N Hassan, Ahmad Reza Hosseinpoor, Sergio Iavicoli, Jaffar Jandaghi, Dorota I Jarosinska, Spo M Kgalamono, Mona Khaleghy Rad, Mostafa Khodabakshi, Xinxin Li, Alessandro Marinaccio, Guy Mbayo, Zohreh Rowshani, Natasha M Sanabria, Kerry Sidwell-Wilson, Orielle H Solar, Kai N Streicher, Xin Sun, Rahim Taghizadeh Asl, Mehrdad Yadegari, Siyu Zhang, Muzimkhulu Zungu, Natalie C Momen

**Affiliations:** a Department of Environment, Climate Change and Health, World Health Organization, Avenue Appia 20, 1211 Geneva 27, Switzerland.; b Department of Healthier Populations, World Health Organization Regional Office for the Eastern Mediterranean, Amman, Jordan.; c Department of Data and Analytics, World Health Organization, Geneva, Switzerland.; d Climate Change, Health and Environment, World Health Organization Regional Office for Africa, Brazzaville, Congo.; e Division of Healthy Environments and Populations, World Health Organization Regional Office for the Western Pacific, Manila, Philippines.; f Department of Occupational and Environmental Medicine, Epidemiology and Hygiene, Italian National Institute for Insurance against Accidents at Work, Rome, Italy.; g Department of Health Promotion and Social Determinants, Pan American Health Organization, Washington DC, United States of America.; h Department of Healthier Populations and Noncommunicable Diseases, World Health Organization Regional Office for South-East Asia, New Delhi, India.; i Directorate for Communication and European and International Relations, Ministry of Health, Rome, Italy.; j Center for Environmental and Occupational Health, Ministry of Health and Medical Education, Tehran, Islamic Republic of Iran.; k World Health Organization European Centre for Environment and Health, Bonn, Germany.; l National Institute for Occupational Health, Johannesburg, South Africa.; m Healthier Population Unit, World Health Organization Country Office, Tehran, Islamic Republic of Iran.; n National Institute for Occupational Health and Poison Control, Chinese Center for Disease Control and Prevention, Beijing, China.

## Abstract

Through sustainable development goals 3 and 8 and other policies, countries have committed to protect and promote workers’ health by reducing the work-related burden of disease. To monitor progress on these commitments, indicators that capture the work-related burden of disease should be available for monitoring workers’ health and sustainable development. The World Health Organization and the International Labour Organization estimate that only 363 283 (19%) of 1 879 890 work-related deaths globally in 2016 were due to injuries, whereas 1 516 607 (81%) deaths were due to diseases. Most monitoring systems focusing on workers’ health or sustainable development, such as the global indicator framework for the sustainable development goals, include an indicator on the burden of occupational injuries. Few such systems, however, have an indicator on the burden of work-related diseases. To address this gap, we present a new global indicator: mortality rate from diseases attributable to selected occupational risk factors, by disease, risk factor, sex and age group. We outline the policy rationale of the indicator, describe its data sources and methods of calculation, and report and analyse the official indicator for 183 countries. We also provide examples of the use of the indicator in national workers’ health monitoring systems and highlight the indicator’s strengths and limitations. We conclude that integrating the new indicator into monitoring systems will provide more comprehensive and accurate surveillance of workers’ health, and allow harmonization across global, regional and national monitoring systems. Inequalities in workers’ health can be analysed and the evidence base can be improved towards more effective policy and systems on workers’ health.

## Résumé

Par le biais des objectifs de développement durable 3 et 8 ainsi que d'autres mesures, plusieurs pays se sont engagés à protéger et promouvoir la santé des travailleurs en réduisant l'impact des maladies liées au travail. Mais pour évaluer leurs progrès en la matière, il convient de mettre en place des indicateurs estimant l'impact des maladies liées au travail afin de placer le développement durable et la santé des travailleurs sous surveillance. D'après l'Organisation mondiale de la Santé et l'Organisation internationale du Travail, seulement 363 283 (19%) des 1 879 890 décès liés au travail dans le monde en 2016 découlaient de blessures, tandis que 1 516 607 (81%) d'entre eux étaient causés par des maladies. La plupart des systèmes de surveillance qui s'intéressent à la santé des travailleurs ou au développement durable, comme le cadre mondial d'indicateurs pour les objectifs de développement durable, comportent un indicateur relatif à l'impact des accidents de travail. Cependant, rares sont ceux qui possèdent un indicateur concernant l'impact des maladies professionnelles. Pour combler cette lacune, nous dévoilons un nouvel indicateur mondial: le taux de mortalité dû aux maladies attribuables à certains facteurs de risque professionnels classé par maladie, facteur de risque, sexe et catégorie d'âge. Nous exposons le motif politique de l'indicateur, décrivons l'origine des données et les méthodes de calcul, et communiquons et analysons l'indicateur officiel pour 183 pays. Nous fournissons également des exemples de la façon dont l'indicateur peut être utilisé dans des systèmes nationaux de surveillance de la santé des travailleurs et soulignons ses forces et faiblesses. Nous concluons en affirmant que l'intégration de ce nouvel indicateur dans les systèmes de surveillance offrira un suivi plus complet et précis de la santé des travailleurs et ouvrira la voie à une harmonisation des systèmes mondiaux, nationaux et régionaux. Il est possible d'analyser les inégalités en matière de santé des travailleurs et d'en améliorer les bases factuelles afin d'établir des politiques et systèmes plus efficaces dans ce domaine.

## Resumen

A través de los objetivos de desarrollo sostenible 3 y 8 y de otras políticas, los países se han comprometido a proteger y promover la salud de los trabajadores reduciendo la carga de morbilidad relacionada con el trabajo. Para supervisar los avances en el cumplimiento de estos compromisos, debería disponerse de indicadores que reflejen la carga de morbilidad relacionada con el trabajo, a fin de controlar la salud de los trabajadores y el desarrollo sostenible. La Organización Mundial de la Salud y la Organización Internacional del Trabajo estiman que solo 363 283 (19%) de las 1 879 890 muertes relacionadas con el trabajo a nivel mundial en 2016 se debieron a lesiones, mientras que 1 516 607 (81%) muertes se debieron a enfermedades. La mayoría de los sistemas de vigilancia centrados en la salud de los trabajadores o el desarrollo sostenible, como el marco de indicadores mundiales para los objetivos de desarrollo sostenible, incluyen un indicador sobre la carga de las lesiones laborales. No obstante, pocos de estos sistemas cuentan con un indicador sobre la carga de las enfermedades relacionadas con el trabajo. Para subsanar esta carencia, presentamos un nuevo indicador mundial: la tasa de mortalidad por enfermedades atribuibles a factores de riesgo laborales seleccionados, por enfermedad, factor de riesgo, sexo y grupo de edad. Describimos la justificación política del indicador, describimos sus fuentes de datos y métodos de cálculo, e informamos y analizamos el indicador oficial para 183 países. También proporcionamos ejemplos del uso del indicador en los sistemas nacionales de vigilancia de la salud de los trabajadores y destacamos las ventajas y las limitaciones del indicador. Concluimos que la integración del nuevo indicador en los sistemas de vigilancia proporcionará una vigilancia más exhaustiva y precisa de la salud de los trabajadores, y permitirá la armonización entre los sistemas de vigilancia mundiales, regionales y nacionales. Se podrán analizar las desigualdades en la salud de los trabajadores y se podrá mejorar la base de evidencias para lograr políticas y sistemas más eficaces en materia de salud de los trabajadores.

## ملخص

من خلال هدفي التنمية المستدامة 3 و8، والسياسات الأخرى، التزمت الدول بحماية وتعزيز صحة العمال عن طريق الحد من عبء المرض المرتبط بالعمل. لمراقبة التقدم الذي تم تحقيقه في هذه الالتزامات، يجب أن تكون المؤشرات، التي ترصد عبء المرض المرتبط بالعمل، متاحة لرصد صحة العمال والتنمية المستدامة. تقدر كل من منظمة الصحة العالمية، ومنظمة العمل الدولية، أن 363283 (%19) فقط من إجمالي 18879890 من حالات الوفاة، مرتبطة بالعمل على مستوى العالم في عام 2016، كانت بسبب الإصابات، في حين أن 1516607 (%81) من حالات الوفاة كانت بسبب الأمراض. معظم أنظمة المراقبة التي تركز على صحة العمال أو التنمية المستدامة، مثل إطار عمل المؤشر العالمي لأهداف التنمية المستدامة، تتضمن مؤشرًا على عبء الإصابات المهنية. ومع ذلك، فإن القليل من هذه الأنظمة لديها مؤشر على عبء الأمراض المرتبطة بالعمل. للتعامل مع هذه الفجوة، نحن نقدم مؤشرًا عالميًا جديدًا: معدل الوفيات المرتبطة الأمراض الناجمة عن عوامل خطر مهنية محددة، حسب المرض، وعامل الخطر، والجنس، والفئة العمرية. نحن نحدد الأساس المنطقي لسياسة المؤشر، ونصف مصادر بياناته وطرق حسابه، ونعلن عن، ونحلل المؤشر الرسمي لـ 183 دولة. نحن نقدم أيضًا أمثلة لاستخدام المؤشر في أنظمة مراقبة صحة العمال الوطنية، ونركز على نقاط القوة والقيود الخاصة بالمؤشر. نحن نستنتج أن دمج المؤشر الجديد في أنظمة المراقبة سيوفر مراقبة أكثر شمولاً ودقة لصحة العمال، ويسمح بالتنسيق عبر أنظمة المراقبة العالمية والإقليمية والوطنية. يمكن تحليل حالات التفاوت في صحة العمال، ويمكن تحسين قاعدة الأدلة نحو تحقيق سياسة وأنظمة أكثر فعالية بالنسبة لصحة العمال.

## 摘要

通过可持续发展目标 3 和 8 以及其他政策，各国承诺通过减少与工作相关的疾病负担来保护和促进工人健康。为了监测这些承诺的进展情况，应当提供反映与工作相关的疾病负担的指标，以用于监测工人的健康和可持续发展。据世界卫生组织和国际劳工组织估计，2016 年全球 1,879,890 例与工作相关的死亡中，只有 363,283 例 (19%) 是因工伤死亡，而1,516,607 例 (81%) 是死于疾病。大多数关注工人健康或可持续发展的监测系统，如可持续发展目标的全球指标框架，都包括一项关于职业伤害负担的指标。然而，这些系统中很少有关于与工作有关的疾病负担的指标。为了弥补这一差距，我们提出了一个全球性的新指标：按疾病、风险因素、性别和年龄组划分的可归因于选定职业风险因素的疾病死亡率。我们概述了该指标的政策依据，描述了其数据来源和计算方法，并报告和分析了 183 个国家/地区的官方指标。我们还提供了在国家工人健康监测系统中使用该指标的例子，并强调了该指标的优势和局限性。我们的结论是，将新指标纳入监测系统将提供更全面和准确的工人健康监测，并促进了全球、区域和国家监测系统之间的协调性。可以分析工人健康方面的不平等现象，并完善证据基础，以制定更有效的工人健康政策和制度。

## Резюме

В рамках целей в области устойчивого развития 3 и 8 и других стратегий страны обязались защищать и укреплять здоровье работников путем снижения бремени заболеваний, связанных с работой. Для мониторинга прогресса в выполнении этих обязательств необходимо предоставить показатели, отражающие бремя заболеваний, связанных с работой, для мониторинга здоровья работников и устойчивого развития. По оценкам Всемирной организации здравоохранения и Международной организации труда, из 1 879 890 смертей на производстве в мире в 2016 году только 363 283 (19%) были вызваны травмами, тогда как 1 516 607 (81%) смертей были вызваны заболеваниями. Большинство систем мониторинга, ориентированных на здоровье работников или устойчивое развитие, таких как глобальная система показателей для целей в области устойчивого развития, включают показатель нагрузки производственного травматизма. Однако лишь немногие такие системы имеют показатель бремени заболеваний, связанных с работой. Для устранения этого пробела представлен новый глобальный показатель: уровень смертности от заболеваний, связанных с отдельными профессиональными факторами риска, с разбивкой по заболеваниям, факторам риска, полу и возрастным группам. Мы излагаем обоснование показателя в соответствии с политикой организации, описываем источники данных и методы его расчета, а также представляем отчет и анализируем официальный показатель по 183 странам. Также приведены примеры использования показателя в национальных системах мониторинга здоровья работников, и отмечены сильные и слабые стороны показателя. Мы пришли к выводу, что включение нового показателя в системы мониторинга обеспечит более комплексное и точное наблюдение за здоровьем работников и позволит гармонизировать глобальные, региональные и национальные системы мониторинга. Неравенство в здоровье работников может быть проанализировано, а доказательная база может быть улучшена в направлении более эффективной политики и систем в области здоровья работников.

## Introduction

Countries have made policy commitments through the sustainable development goals (SDGs) to ensure health and promote decent work for all.^1^ SDG targets 3.9 and 8.8 are to “substantially reduce the number of deaths and illnesses from hazardous chemicals and air, water and soil pollution and contamination” and “promote safe and secure working environments for all workers.” Countries have committed to workers’ health through the World Health Organization (WHO) *Strategy on health, environment and climate change*;^2^ the *Pan American Health Organization plan of action on workers’ health 2015–2025*;^3^ *International Labour standards on occupational safety and health*;^4^ and the International Labour Organization (ILO) *Framework of fundamental principles and rights at work*.^5^

Achieving these targets requires preventing exposure to occupational risk factors, and reducing the burden of disease that is attributable to unhealthy and indecent working conditions. To track progress, the work-related burden of disease must be monitored in global, regional and national systems for surveillance of workers’ health and progress on sustainable development.

Health indicators are “summary measures that capture relevant information on different attributes and dimensions of health status.”^3^ Indicators of workers’ health have received little research and policy attention, despite their importance in the health, labour and economic development sectors.^6^^,^^7^ The only such indicator commonly included in official monitoring systems is occupational injuries. The global indicator framework for the SDGs tracks the burden of injuries with indicator SDG 8.8.1 (fatal and non-fatal occupational injuries per 100 000 workers, by sex and migrant status).^8^^,^^9^ Data on fatal occupational injuries for the indicator were reported by 35 countries for 2016.

In 2021, WHO and the ILO published the first WHO/ILO joint estimates of the work-related burden of disease and injury.^10^^–^^13^ According to these estimates for 2016, only 363 283 (19%) out of 1 879 890 global work-related deaths were due to injuries, while the majority, 1 516 607 (81%), were due to work-related diseases.^10^ To assess progress towards agreed targets for workers’ health, the burden of mortality from work-related diseases also needs to be tracked. WHO’s Thirteenth General Programme of Work, approved by the Seventy-First World Health Assembly of the 194 WHO Member States,^14^ mandates WHO to promote and improve the monitoring of work-related diseases.^15^

**Fig. 1:**
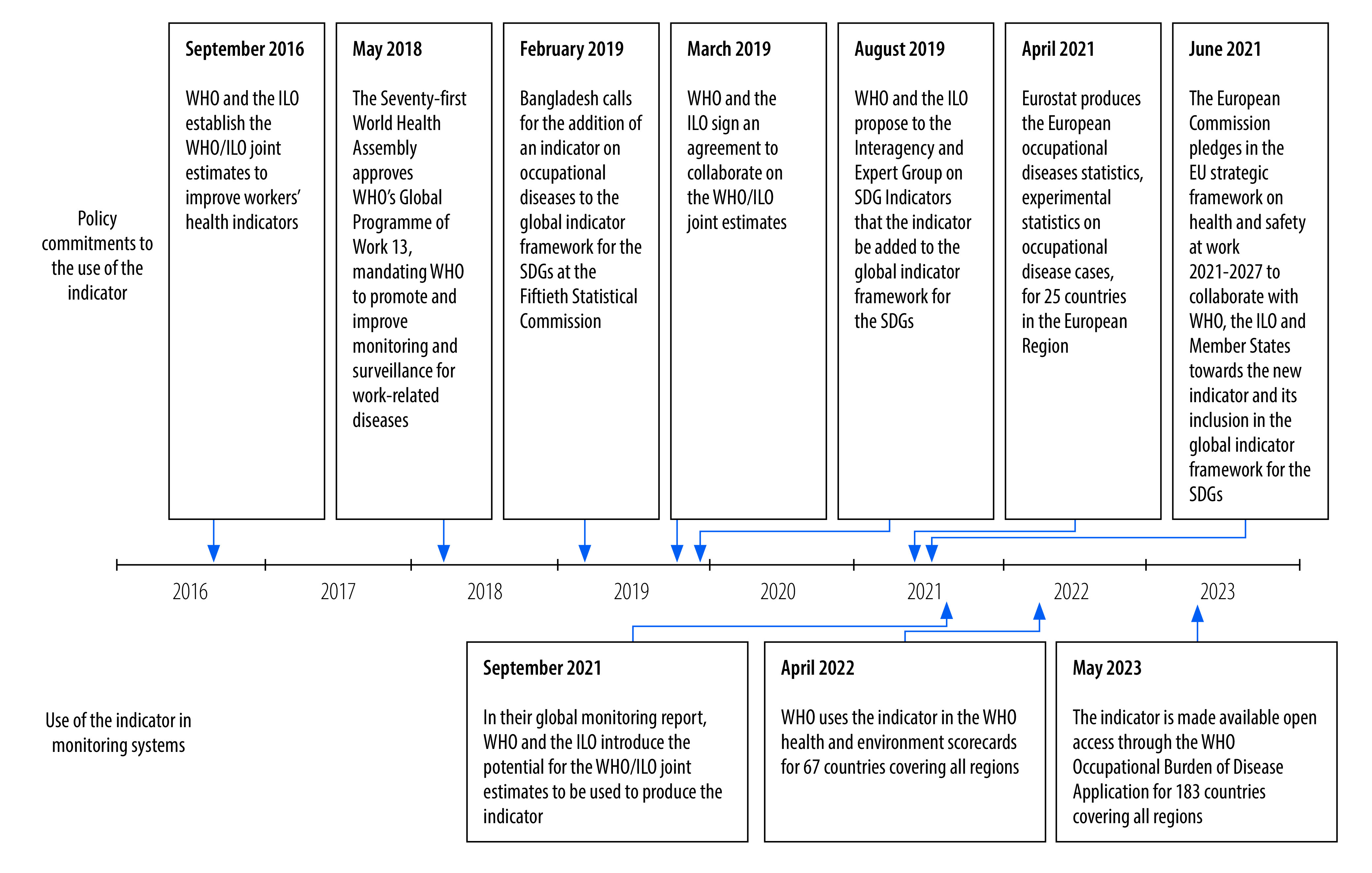
Timeline of policy commitments to the new global indicator for work-related burden of disease

ILO: International Labour Organization; SDGs: sustainable development goals; WHO: World Health Organization.

To address this gap, we describe a new global indicator: mortality rate from diseases attributable to selected occupational risk factors, by disease, risk factor, sex and age group. The need for such an indicator has been recognized for several years (Fig. 1). In 2019, WHO and the ILO proposed that an indicator be added to the global indicator framework for the SDGs^9^ to capture deaths from work-related diseases (WHO and the ILO, Proposal: indicator 8.8.3: mortality rate from diseases attributed to occupational risk factors, by disease, risk factor, sex, and age group: submission to the Interagency and Expert Group on SDG Indicators, personal communication, 2019). We outline the methods and data sources for calculating this indicator from the WHO/ILO joint estimates. We also report the indicator calculated for 183 countries, and apply it to analyse the global and regional epidemiology and socioeconomic inequalities of fatal work-related diseases. Additionally, we present country case studies on the integration of the indicator into national workers’ health monitoring systems, and highlight the strengths and limitations of the tool.

## Calculating the indicator

### Data sources

To calculate the indicator (the mortality rate), we required two data sets: the number of deaths from diseases attributable to selected occupational risk factors (the numerator); and the working-age population, defined here as age 15 years and older (the denominator).^10^^,^^11^ We obtained these data for population cohorts defined by country (183 countries), region (six WHO Regions), sex (female, male, total) and age (≥ 15, 15–19, 20–24, …, 90–94, ≥ 95 years) in the years 2000, 2010 and 2016.

We sourced the number of deaths from diseases attributable to selected occupational risk factors from the United Nations’ official estimates of the work-related burden of disease: the WHO/ILO joint estimates.^10^^–^^13^^,^^16^^,^^17^ These data comprise estimates of the burden attributable to 21 pairs of occupational risk factor and disease (Table 1). These exposure–disease pairings are those for which evidence has been systematically reviewed and judged by WHO and the ILO to be sufficient to produce official estimates that fulfil the strict statistical requirements of both organizations.^10^^–^^13^^,^^18^ All diseases covered are noncommunicable diseases within three groups: malignant neoplasms (17 exposure–disease pairs); respiratory diseases (two pairs); and cardiovascular diseases (two pairs).^10^ We downloaded the data from the WHO Occupational Burden of Disease Application.^19^

**Table 1:** Pairings of occupational risk factors and diseases included in the new global indicator for work-related burden of disease

Pair no.	Risk factor	Disease group^c^
1	Occupational exposure to asbestos^a^	Trachea, bronchus and lung cancers^d^
2	Occupational exposure to asbestos^a^	Ovary cancer^d^
3	Occupational exposure to asbestos^a^	Larynx cancer^d^
4	Occupational exposure to asbestos^a^	Mesothelioma^d^
5	Occupational exposure to arsenic^a^	Trachea, bronchus and lung cancers^d^
6	Occupational exposure to benzene^a^	Leukaemia
7	Occupational exposure to beryllium^a^	Trachea, bronchus and lung cancers^d^
8	Occupational exposure to cadmium^a^	Trachea, bronchus and lung cancers^d^
9	Occupational exposure to chromium^a^	Trachea, bronchus and lung cancers^d^
10	Occupational exposure to diesel engine exhaust^a^	Trachea, bronchus and lung cancers^d^
11	Occupational exposure to formaldehyde^a^	Nasopharynx cancer^d^
12	Occupational exposure to formaldehyde^a^	Leukaemia^d^
13	Occupational exposure to nickel^a^	Trachea, bronchus and lung cancers^d^
14	Occupational exposure to polycyclic aromatic hydrocarbons^a^	Trachea, bronchus and lung cancers^d^
15	Occupational exposure to silica^a^	Trachea, bronchus and lung cancers^d^
16	Occupational exposure to sulphuric acid^a^	Larynx cancer^d^
17	Occupational exposure to trichloroethylene^a^	Kidney cancer^d^
18	Occupational asthmagens^a^	Asthma^e^
19	Occupational particulate matter, gases and fumes^a^	Chronic obstructive pulmonary disease^e^
20	Exposure to long working hours^b^	Stroke^f^
21	Exposure to long working hours^b^	Ischaemic heart disease^f^

^a^ Defined as per the classification of the 2017 Global Burden of Disease Study.^16^

^b^ Defined as per World Health Organization (WHO) and International Labour Organization (ILO) joint estimates of the work-related burden of disease and injury definition of ≥ 55 hours per week.^12^

^c^ Defined as per WHO burden of disease classification.^17^

^d^ Malignant neoplasm.^17^

^e^ Respiratory disease.^17^

^f^ Cardiovascular disease.^17^

The data sources and methods for the WHO/ILO joint estimates are described elsewhere,^11^ but these estimates are produced within an established methodological framework: the comparative risk assessment.^20^ This framework results in estimates of work-related burden of disease that can be compared across diseases, risk factors, geographical locations and population cohorts. Countries have approved several SDG indicators produced under WHO guardianship from the comparative risk assessment framework.^9^ All estimates adjust for occupational turnover using WHO-approved methods: for example, estimates for recently added pairs^12^ model workers moving between employment and unemployment or retirement using longitudinal occupation data.^21^ Briefly, for the 19 established exposure–disease pairs (pairs number 1–19 in Table 1) WHO and the ILO derived population-attributable fractions from the 2017 Global Burden of Disease Study.^16^ These fractions quantify the proportion of deaths from a particular disease attributable to a specific risk factor. For two additional exposure–disease pairs (pairs number 20 and 21 in Table 1), WHO and the ILO calculated the population-attributable fractions based on estimates of prevalence of exposure to long working hours produced from 2324 national official surveys conducted in 154 countries,^12^ and pooled risk ratios from WHO/ILO systematic reviews and meta-analyses.^22^^–^^24^ For all 21 exposure–disease pairings, the population-attributable fractions were then applied to the WHO global health estimates of the total number of deaths from each disease^25^ to produce the number of work-related deaths.

We sourced the denominator, total population of working age (≥ 15 years), from the United Nations’ official population estimates: the 2019 Revision of World Population Prospects.^26^

### Calculation method 

We calculated the total number of deaths from diseases attributable to selected occupational risk factors by summing the estimates for the 21 individual exposure–disease pairs (Table 1). We then divided this number of deaths by the total working-age population. We calculated the indicator for national, regional and global population cohorts by sex and age group, using the following formula:
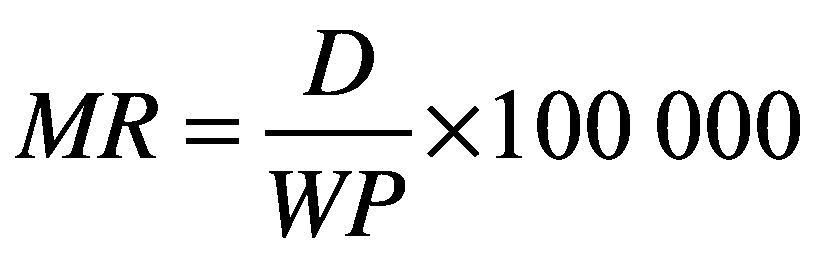
(1)where *MR* is the mortality rate per 100 000 working-age population [disease, occupational risk factor, country, sex, age], *D* is the number of deaths [disease, occupational risk factor, country, sex, age] and *WP* is the number of working-age population [country, sex, age]. This calculation gave the final indicator: mortality rate per 100 000 working-age population from diseases attributable to selected occupational risk factors, by disease, risk factor, sex and age group. For each estimate, we calculated 95% uncertainty ranges (UR) using bootstrapping.^27^

## Applying the indicator

### By region and country

**Fig. 2:**
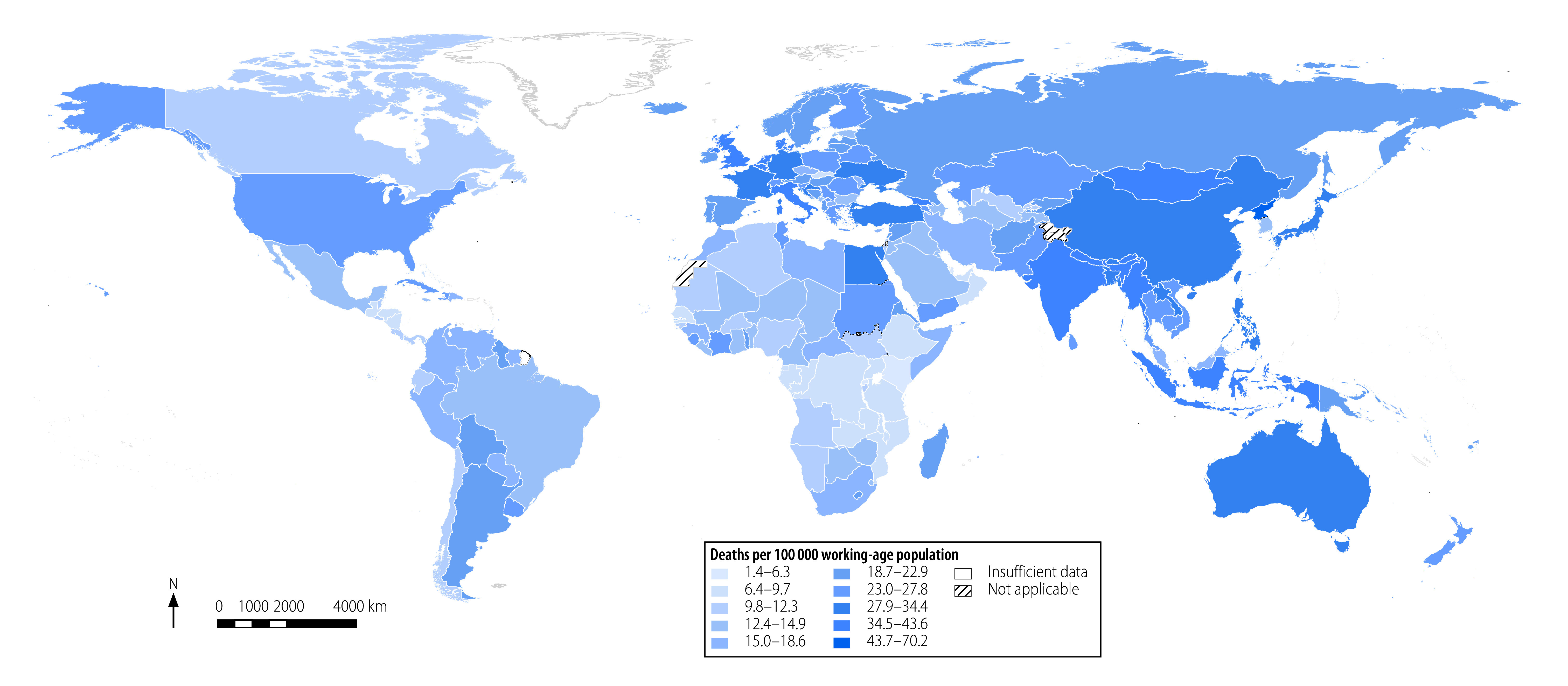
Mortality rate per 100 000 working-age population (≥ 15 years) from diseases attributable to selected occupational risk factors in 183 countries, 2016

Note: Map produced by World Health Organization (WHO) GIS Centre for Health and the Department of Data and Analytics in the Division of Data, Analytics and Delivery for Impact. Map created on26 April 2023.

In Table 2 (available at https://www.who.int/publications/journals/bulletin/) we present the official indicator produced by WHO for 183 countries for the years 2000, 2010 and 2016. The table illustrates how applying the indicator provides a comprehensive picture of the global and regional patterns of mortality from diseases attributable to selected occupational risk factors. The global mortality rate was 27.7 deaths per 100 000 working-age population for 2016 (95% UR: 26.8 to 28.5). Regional mortality rates were highest in the South-East Asia Region (36.5 deaths per 100 000 working-age population; 95% UR: 34.3 to 38.8) and the Western Pacific Region (32.2 deaths per 100 000; 95% UR: 30.3 to 34.2), followed by the European Region (27.3 deaths per 100 000; 95% UR: 26.9 to 27.8; Table 2). Regional mortality rates were lowest in the African Region (11.4 deaths per 100 000; 95% UR: 11.1 to 11.7), followed by the Region of the Americas (18.1 deaths per 100 000; 95% UR: 17.7 to 18.5) and the Eastern Mediterranean Region (21.8 deaths per 100 000; 95% UR: 20.7 to 23.0). Fig. 2 maps the mortality rate in the 183 countries for the year 2016.

**Table 2:** Global, regional and national mortality rate per 100 000 working-age population (≥ 15 years) from diseases attributable to selected occupational risk factors in 183 countries for years 2000, 2010 and 2016

Region, country or area name	Values for the indicator by year												% change (95% UR) of the indicator between years		
	2000				2010				2016				2000–2010	2010–2016	2000–2016
	No. of deaths from work-related diseases (95% UR)^a^	Working-age population, thousands^b^	Deaths per 100 000 population (95% UR)		No. of deaths from work-related diseases (95% UR)^a^	Working-age population, thousands^b^	Deaths per 100 000 population (95% UR)		No. of deaths from work-related diseases (95% UR)^a^	Working-age population, thousands^b^	Deaths per 100 000 population (95% UR)				
**Global**	1 306 102 (1 271 165 to 1 341 039)	4 261 494	30.7 (29.8 to 31.5)		1 405 761 (1 368 657 to 1 442 865)	5 045 358	27.9 (27.1 to 28.6)		1 516 607 (1 472 141 to 1 561 073)	5 484 278	27.7 (26.8 to 28.5)		−9.1 (−12.4 to −5.6)	−0.8 (−4.7 to 3.2)	−9.8 (−13.3 to −6.1)
**WHO Region**															
African Region	51 514 (50 258 to 52 770)	370 807	13.9 (13.6 to 14.2)		58 348 (56 931 to 59 765)	489 005	11.9 (11.6 to 12.2)		66 343 (64 567 to 68 119)	580 925	11.4 (11.1 to 11.7)		−14.1 (−17.0 to −11.1)	−4.3 (−7.8 to −0.9)	−17.8 (−20.8 to −14.8)
Region of the Americas	129 582 (126 659 to 132 505)	595 277	21.8 (21.3 to 22.3)		131 592 (128 927 to 134 257)	698 613	18.8 (18.5 to 19.2)		137 305 (134 429 to 140 181)	758 942	18.1 (17.7 to 18.5)		−13.5 (−16.0 to −10.8)	−4.0 (−6.7 to −1.1)	−16.9 (−19.4 to −14.2)
Eastern Mediterranean Region	73 809 (70 185 to 77 433)	286 589	25.8 (24.5 to 27.0)		87 877 (83 495 to 92 259)	386 987	22.7 (21.6 to 23.8)		97 727 (92 663 to 102 791)	447 609	21.8 (20.7 to 23.0)		−11.8 (−17.8 to −5.4)	−3.9 (−10.6 to 3.4)	−15.2 (−21.2 to −9.0)
European Region	216 022 (211 236 to 220 808)	693 894	31.1 (30.4 to 31.8)		212 083 (208 115 to 216 051)	738 503	28.7 (28.2 to 29.3)		206 219 (202 701 to 209 737)	754 316	27.3 (26.9 to 27.8)		−7.8 (−10.4 to −5.0)	−4.8 (−7.2 to −2.3)	−12.2 (−14.6 to −9.7)
South-East Asia Region	374 562 (353 500 to 395 624)	1 042 010	36.0 (33.9 to 38.0)		449 732 (423 652 to 475 812)	1 268 881	35.4 (33.4 to 37.5)		514 564 (482 571 to 546 557)	1 408 816	36.5 (34.3 to 38.8)		−1.4 (−9.1 to 6.8)	3.1 (−5.3 to 12.5)	1.6 (−6.6 to 10.3)
Western Pacific Region	460 613 (433 579 to 487 647)	1 272 917	36.2 (34.1 to 38.3)		466 129 (440 585 to 491 673)	1 463 370	31.9 (30.1 to 33.6)		494 449 (464 379 to 524 519)	1 533 668	32.2 (30.3 to 34.2)		−12 (−18.7 to −4.5)	1.2 (−6.8 to 9.7)	−10.9 (−18.1 to −3.0)
**Countries**															
Afghanistan	3 047 (2 691 to 3 403)	10 620	28.7 (25.3 to 32.0)		3 887 (3 428 to 4 346)	15 124	25.7 (22.7 to 28.7)		4 382 (3 841 to 4 923)	19 718	22.2 (19.5 to 25.0)		−10.4 (−24.2 to 5.9)	−13.5 (−27.2 to 2.4)	−22.5 (−34.9 to −7.9)
Albania	381 (332 to 430)	2 180	17.5 (15.2 to 19.8)		451 (393 to 509)	2 286	19.7 (17.2 to 22.3)		487 (426 to 548)	2 356	20.7 (18.1 to 23.3)		12.9 (−5.6 to 35.9)	4.8 (−12.2 to 25.3)	18.3 (−1.2 to 42.2)
Algeria	3 217 (2 842 to 3 592)	20 375	15.8 (14.0 to 17.6)		3 020 (2 684 to 3 356)	26 162	11.5 (10.3 to 12.8)		3 331 (2 953 to 3 709)	28 737	11.6 (10.3 to 12.9)		−26.9 (−37.7 to −14.0)	0.4 (−14.9 to 17.9)	−26.6 (−37.7 to −13.4)
Angola	1 214 (1 068 to 1 360)	8 657	14.0 (12.3 to 15.7)		1 310 (1 152 to 1 468)	12 369	10.6 (9.3 to 11.9)		1 584 (1 378 to 1 790)	15 271	10.4 (9.0 to 11.7)		−24.5 (−36.6 to −10.4)	−2.1 (−18.3 to 16.7)	−26.0 (−38.3 to −11.8)
Antigua and Barbuda	2 (0 to 5)	54	3.7 (0.0 to 8.4)		2 (0 to 5)	67	3.0 (0.0 to 7.0)		1 (0 to 4)	73	1.4 (0.0 to 2.7)		−18.6 (−117.7 to 435.1)	−54.6 (−156.5 to 183.2)	−63.1 (−178.1 to 191.3)
Argentina	7 439 (6 907 to 7 971)	26 377	28.2 (26.2 to 30.2)		7 558 (7 114 to 8 002)	30 248	25.0 (23.5 to 26.5)		6 991 (6 587 to 7 395)	32 597	21.5 (20.2 to 22.7)		−11.4 (−19.1 to −2.7)	−14.2 (−20.9 to −6.7)	−24.0 (−30.6 to −16.6)
Armenia	596 (542 to 650)	2 277	26.2 (23.8 to 28.5)		627 (579 to 675)	2 317	27.1 (25.0 to 29.1)		590 (545 to 635)	2 341	25.2 (23.3 to 27.1)		3.4 (−8.1 to 16.6)	−6.9 (−16.3 to 3.7)	−3.7 (−14.5 to 8.6)
Australia	5 195 (4 932 to 5 458)	15 026	34.6 (32.8 to 36.3)		5 434 (5 207 to 5 661)	17 938	30.3 (29.0 to 31.6)		5 604 (5 361 to 5 847)	19 664	28.5 (27.3 to 29.7)		−12.4 (−18.0 to −6.2)	−5.9 (−11.5 to −0.1)	−17.6 (−22.8 to −11.9)
Austria	1 639 (1 532 to 1 746)	6 711	24.4 (22.8 to 26.0)		1 698 (1 611 to 1 785)	7 172	23.7 (22.5 to 24.9)		1 719 (1 627 to 1 811)	7 508	22.9 (21.7 to 24.1)		−3.1 (−10.7 to 5.5)	−3.3 (−10.1 to 4.3)	−6.2 (−13.7 to 1.9)
Azerbaijan	998 (875 to 1 121)	5 593	17.8 (15.6 to 20.1)		985 (873 to 1 097)	6 970	14.1 (12.5 to 15.7)		1 013 (896 to 1 130)	7 483	13.5 (12.0 to 15.1)		−20.8 (−33.1 to −6.1)	−4.2 (−19.0 to 12.6)	−24.1 (−36.2 to −10.0)
Bahamas	14 (10 to 18)	211	6.6 (4.9 to 8.4)		19 (15 to 23)	260	7.3 (5.7 to 9.0)		20 (15 to 25)	290	6.9 (5.4 to 8.5)		10.2 (−18.5 to 47.7)	−5.7 (−32.0 to 31.8)	3.9 (−24.7 to 42.3)
Bahrain	42 (34 to 50)	464	9.1 (7.4 to 10.7)		52 (44 to 60)	989	5.3 (4.5 to 6.1)		55 (46 to 64)	1135	4.9 (4.1 to 5.6)		−41.9 (−54.0 to −25.4)	−7.8 (−26.2 to 15.2)	−46.4 (−57.7 to −31.8)
Bangladesh	21 657 (19 726 to 23 588)	80 479	26.9 (24.5 to 29.3)		31 059 (28 135 to 33 983)	100 371	30.9 (28.0 to 33.9)		36 260 (32 902 to 39 618)	112 554	32.2 (29.2 to 35.2)		15.0 (0.6 to 31.0)	4.1 (−8.8 to 18.7)	19.7 (5.0 to 36.0)
Barbados	33 (26 to 40)	212	15.5 (12.3 to 18.8)		30 (24 to 36)	226	13.3 (10.5 to 16.0)		31 (25 to 37)	234	13.2 (10.6 to 15.9)		−14.8 (−35.5 to 12.7)	−0.2 (−24.8 to 32.3)	−15.0 (−36.3 to 11.6)
Belarus	3 219 (2 891 to 3 547)	8 043	40.0 (36.0 to 44.1)		2 605 (2 305 to 2 905)	8017	32.5 (28.8 to 36.2)		1 899 (1 666 to 2 132)	7 886	24.1 (21.1 to 27.0)		−18.8 (−30.6 to −5.3)	−25.9 (−37.5 to −12.1)	−39.8 (−48.9 to −29.6)
Belgium	3 626 (3 448 to 3 804)	8 476	42.8 (40.7 to 44.9)		3 757 (3 582 to 3 932)	9 092	41.3 (39.4 to 43.2)		3 454 (3 278 to 3 630)	9 422	36.7 (34.8 to 38.5)		−3.4 (−9.8 to 3.3)	−11.3 (−17.2 to −5.1)	−14.3 (−20.1 to −8.2)
Belize	21 (17 to 25)	147	14.3 (11.3 to 17.3)		22 (17 to 27)	208	10.6 (8.3 to 12.9)		26 (20 to 32)	252	10.3 (8.3 to 12.4)		−26.1 (−45.3 to −1.5)	−2.7 (−27.9 to 33.3)	−28.1 (−46.1 to −4.7)
Benin	548 (493 to 603)	3 767	14.6 (13.1 to 16.0)		737 (665 to 809)	5 166	14.3 (12.9 to 15.7)		922 (823 to 1 021)	6 212	14.8 (13.3 to 16.4)		−1.9 (−15.0 to 13.2)	4.0 (−9.7 to 20.3)	2.0 (−12.1 to 18.4)
Bhutan	139 (124 to 154)	356	39.1 (34.8 to 43.4)		150 (134 to 166)	472	31.8 (28.4 to 35.2)		163 (144 to 182)	540	30.2 (26.8 to 33.6)		−18.6 (−30.1 to −4.8)	−5.0 (−18.7 to 11.0)	−22.7 (−34.1 to −9.7)
Bolivia (Plurinational State of)	1 448 (1 310 to 1 586)	5 241	27.6 (25.0 to 30.3)		1 390 (1 259 to 1 521)	6 583	21.1 (19.1 to 23.1)		1 472 (1 328 to 1 616)	7 503	19.6 (17.7 to 21.5)		−23.6 (−33.4 to −12.3)	−7.1 (−18.8 to 6.5)	−29.0 (−38.0 to −18.4)
Bosnia and Herzegovina	718 (639 to 797)	2 974	24.1 (21.5 to 26.8)		695 (628 to 762)	3 122	22.3 (20.1 to 24.4)		603 (547 to 659)	2 881	20.9 (19.0 to 22.9)		−7.8 (−20.2 to 6.9)	−6.0 (−17.7 to 7.6)	−13.3 (−24.7 to 0.5)
Botswana	138 (119 to 157)	1 008	13.7 (11.8 to 15.6)		178 (155 to 201)	1 292	13.8 (12.0 to 15.6)		196 (168 to 224)	1 410	13.9 (11.9 to 15.9)		0.6 (−16.0 to 21.5)	0.9 (−16.7 to 22.7)	1.5 (−16.9 to 23.5)
Brazil	20 990 (19 496 to 22 484)	122 461	17.1 (15.9 to 18.4)		20 736 (19 394 to 22 078)	147 131	14.1 (13.2 to 15.0)		20 844 (19 560 to 22 128)	160 705	13.0 (12.2 to 13.8)		−17.8 (−25.4 to −9.5)	−8.0 (−15.7 to 0.7)	−24.3 (−31.1 to −16.7)
Brunei Darussalam	15 (11 to 19)	231	6.5 (5.0 to 8.0)		21 (17 to 25)	288	7.3 (5.8 to 8.8)		32 (26 to 38)	320	10.0 (8.3 to 11.8)		12.4 (−14.7 to 52.9)	37.2 (7.0 to 76.9)	54.3 (16.2 to 108.6)
Bulgaria	1 009 (916 to 1 102)	6 745	15.0 (13.6 to 16.3)		954 (887 to 1 021)	6423	14.9 (13.8 to 15.9)		856 (799 to 913)	6 128	14.0 (13.0 to 14.9)		−0.7 (−11.6 to 11.9)	−6.0 (−15.0 to 3.6)	−6.6 (−16.6 to 4.8)
Burkina Faso	740 (649 to 831)	6 179	12.0 (10.5 to 13.4)		914 (803 to 1 025)	8 392	10.9 (9.6 to 12.2)		1 073 (941 to 1 205)	10 183	10.5 (9.2 to 11.8)		−9.1 (−23.8 to 8.9)	−3.3 (−18.5 to 15.4)	−12.0 (−26.1 to 5.0)
Burundi	446 (390 to 502)	3185	14.0 (12.2 to 15.8)		598 (532 to 664)	4 759	12.6 (11.2 to 13.9)		702 (621 to 783)	5 709	12.3 (10.9 to 13.7)		−10.3 (−24.1 to 6.7)	−2.1 (−16.4 to 14.8)	−12.2 (−26 to 4.0)
Cabo Verde	43 (35 to 51)	244	17.6 (14.5 to 20.7)		47 (38 to 56)	333	14.1 (11.5 to 16.7)		40 (33 to 47)	374	10.7 (8.9 to 12.5)		−20.0 (−38.6 to 4.2)	−24.1 (−40.5 to −2.0)	−39.3 (−53.1 to −21.7)
Cambodia	2 012 (1 817 to 2 207)	7 100	28.3 (25.6 to 31.1)		2322 (2 101 to 2 543)	9 542	24.3 (22.0 to 26.7)		2 586 (2 332 to 2 840)	10 817	23.9 (21.6 to 26.3)		−14.1 (−25.0 to −1.4)	−1.8 (−14.6 to 13.0)	−15.6 (−26.6 to −3.1)
Cameroon	1 109 (1 002 to 1 216)	8519	13.0 (11.8 to 14.3)		1 387 (1 254 to 1 520)	11 454	12.1 (11.0 to 13.3)		1 777 (1 592 to 1962)	13 623	13.0 (11.7 to 14.4)		−7.0 (−19.1 to 7.0)	7.7 (−6.6 to 24.0)	0.2 (−13.2 to 15.4)
Canada	7 661 (7 266 to 8 056)	24 727	31.0 (29.4 to 32.6)		8 182 (7 808 to 8 556)	28 517	28.7 (27.4 to 30.0)		8 507 (8 098 to 8 916)	30 599	27.8 (26.5 to 29.1)		−7.4 (−13.5 to −0.6)	−3.1 (−9.4 to 3.5)	−10.3 (−16.3 to −3.6)
Central African Republic	427 (374 to 480)	2 065	20.7 (18.1 to 23.3)		449 (395 to 503)	2 454	18.3 (16.1 to 20.5)		391 (339 to 443)	2498	15.7 (13.6 to 17.7)		−11.5 (−25.6 to 5.6)	−14.4 (−28.5 to 2.0)	−24.3 (−37.1 to −9.3)
Chad	769 (685 to 853)	4 272	18.0 (16.0 to 20.0)		979 (877 to 1 081)	6123	16.0 (14.3 to 17.7)		1 115 (989 to 1 241)	7 625	14.6 (13.0 to 16.3)		−11.2 (−23.7 to 3.6)	−8.5 (−21.7 to 6.5)	−18.8 (−30.5 to −4.6)
Chile	1 259 (1 156 to 1 362)	11 149	11.3 (10.4 to 12.2)		1 602 (1 492 to 1 712)	13 295	12.1 (11.2 to 12.9)		1 654 (1 541 to 1 767)	14 513	11.4 (10.6 to 12.2)		6.7 (−4.1 to 18.9)	−5.4 (−14.2 to 4.4)	0.9 (−9.2 to 12.1)
China	387 582 (360 658 to 414 506)	970 640	39.9 (37.2 to 42.7)		373 095 (347 713 to 398 477)	1 113 391	33.5 (31.2 to 35.8)		392 291 (362 400 to 422 182)	1 159 596	33.8 (31.3 to 36.4)		−16.1 (−23.8 to −7.6)	1.0 (−9.0 to 12.0)	−15.3 (−23.6 to −6.1)
Colombia	4 522 (4 113 to 4 931)	26 721	16.9 (15.4 to 18.5)		5 199 (4 721 to 5 677)	32 891	15.8 (14.4 to 17.3)		5 784 (5 230 to 6 338)	36 591	15.8 (14.3 to 17.3)		−6.6 (−17.9 to 6.5)	0.0 (−12.8 to 14.3)	−6.6 (−18 to 7.0)
Comoros	24 (19 to 29)	304	7.9 (6.3 to 9.5)		25 (19 to 31)	407	6.2 (4.8 to 7.5)		30 (23 to 37)	478	6.3 (5.0 to 7.6)		−22.2 (−42.7 to 5.1)	2.2 (−24.8 to 39.8)	−20.5 (−41.1 to 7.2)
Congo	211 (183 to 239)	1 809	11.7 (10.1 to 13.2)		205 (179 to 231)	2 494	8.2 (7.2 to 9.2)		213 (185 to 241)	2 885	7.4 (6.4 to 8.4)		−29.6 (−41.2 to −15.1)	−10.2 (−25.2 to 7.5)	−36.7 (−47.6 to −23.1)
Costa Rica	338 (304 to 372)	2 728	12.4 (11.1 to 13.6)		384 (345 to 423)	3 465	11.1 (10.0 to 12.2)		415 (371 to 459)	3 829	10.8 (9.7 to 12.0)		−10.6 (−22.5 to 2.8)	−2.2 (−15.7 to 13.4)	−12.5 (−24.2 to 1.1)
Côte d’Ivoire	2 072 (1 843 to 2 301)	9 275	22.3 (19.9 to 24.8)		2914 (2 602 to 3 226)	11 576	25.2 (22.5 to 27.9)		3 406 (3 017 to 3 795)	13 726	24.8 (22.0 to 27.7)		12.7 (−3.6 to 32.0)	−1.4 (−15.7 to 15.6)	11.1 (−5.2 to 30.9)
Croatia	952 (891 to 1 013)	3 661	26.0 (24.3 to 27.7)		1 235 (1 167 to 1 303)	3 660	33.8 (31.9 to 35.6)		1 187 (1 115 to 1 259)	3 601	33.0 (31.0 to 35.0)		29.8 (19.3 to 40.9)	−2.3 (−10.0 to 5.8)	26.8 (16.0 to 38.6)
Cuba	2 077 (1 898 to 2 256)	8 725	23.8 (21.8 to 25.9)		2 311 (2 128 to 2 494)	9 234	25.0 (23.0 to 27.0)		2 379 (2 196 to 2 562)	9 456	25.2 (23.2 to 27.1)		5.1 (−6.4 to 18.1)	0.5 (−10.0 to 12.1)	5.7 (−5.7 to 18.8)
Cyprus	147 (128 to 166)	732	20.1 (17.5 to 22.7)		156 (138 to 174)	914	17.1 (15.1 to 19.1)		169 (146 to 192)	973	17.4 (15.1 to 19.7)		−15.0 (−28.6 to 1.5)	1.8 (−15.1 to 21.6)	−13.5 (−28.6 to 4.2)
Czechia	1 705 (1 586 to 1 824)	8 600	19.8 (18.4 to 21.2)		1 519 (1 428 to 1 610)	9 036	16.8 (15.8 to 17.8)		1 492 (1 408 to 1 576)	9 004	16.6 (15.6 to 17.5)		−15.2 (−22.7 to −7.0)	−1.4 (−9.2 to 7.1)	−16.4 (−23.5 to −8.4)
Democratic People's Republic of Korea	7 567 (6 873 to 8 261)	16 982	44.6 (40.5 to 48.7)		12 914 (11 648 to 14 180)	18 991	68.0 (61.3 to 74.7)		14 084 (12 690 to 15 478)	20 069	70.2 (63.2 to 77.1)		52.6 (33.2 to 74.7)	3.2 (−9.9 to 18.5)	57.5 (38.1 to 80.2)
Democratic Republic of the Congo	2 483 (2 201 to 2 765)	25 654	9.7 (8.6 to 10.8)		2 954 (2 634 to 3 274)	34 774	8.5 (7.6 to 9.4)		3 452 (3 062 to 3 842)	42 273	8.2 (7.2 to 9.1)		−12.2 (−24.9 to 2.6)	−3.9 (−18.0 to 12.7)	−15.6 (−28.1 to −1.1)
Denmark	1 779 (1 694 to 1 864)	4 355	40.9 (38.9 to 42.8)		1 756 (1 672 to 1 840)	4558	38.5 (36.7 to 40.4)		1 820 (1 725 to 1915)	4 763	38.2 (36.2 to 40.2)		−5.7 (−11.9 to 0.8)	−0.8 (−7.5 to 6.5)	−6.5 (−13.1 to 0.4)
Djibouti	52 (42 to 62)	424	12.3 (9.9 to 14.7)		64 (53 to 75)	567	11.3 (9.3 to 13.3)		82 (67 to 97)	647	12.7 (10.5 to 14.9)		−8.0 (−29.3 to 21.1)	12.2 (−13.5 to 45.4)	3.2 (−20.6 to 34.7)
Dominican Republic	842 (730 to 954)	5 514	15.3 (13.2 to 17.3)		1 021 (887 to 1 155)	6 727	15.2 (13.2 to 17.2)		1 182 (1 022 to 1 342)	7 431	15.9 (13.8 to 18.1)		−0.6 (−17.5 to 20.2)	4.8 (−13.2 to 26.4)	4.2 (−14.3 to 26.0)
Ecuador	1 071 (985 to 1 157)	8 247	13 (11.9 to 14.0)		1 065 (983 to 1 147)	10 352	10.3 (9.5 to 11.1)		1 319 (1 211 to 1 427)	11 757	11.2 (10.3 to 12.1)		−20.8 (−29.2 to −11.3)	9.1 (−2.6 to 22.1)	−13.6 (−23.0 to −2.9)
Egypt	11 710 (10 355 to 13 065)	43 462	26.9 (23.8 to 30.1)		15 940 (14 084 to 17 796)	55 817	28.6 (25.2 to 31.9)		18 280 (15 999 to 20 561)	62 670	29.2 (25.5 to 32.8)		6.0 (−10.4 to 25.2)	2.1 (−13.8 to 21.1)	8.3 (−8.8 to 28.6)
El Salvador	465 (411 to 519)	3 731	12.5 (11.0 to 13.9)		427 (378 to 476)	4 227	10.1 (9.0 to 11.3)		461 (409 to 513)	4 586	10.1 (8.9 to 11.2)		−19.0 (−30.9 to −4.5)	−0.5 (−15.1 to 17.1)	−19.4 (−31.3 to −4.8)
Equatorial Guinea	62 (51 to 73)	360	17.2 (14.2 to 20.2)		58 (47 to 69)	580	10.0 (8.1 to 11.9)		67 (55 to 79)	760	8.8 (7.3 to 10.3)		−42.0 (−54.9 to −26.2)	−11.8 (−31.4 to 13.6)	−48.9 (−60.0 to −34.7)
Eritrea	362 (315 to 409)	1 245	29.1 (25.3 to 32.9)		403 (353 to 453)	1 919	21.0 (18.4 to 23.6)		415 (359 to 471)	1 956	21.2 (18.4 to 24.1)		−27.8 (−39.5 to −13.3)	1.0 (−16.0 to 21.1)	−27.1 (−39.6 to −11.6)
Estonia	225 (201 to 249)	1 153	19.5 (17.4 to 21.6)		171 (154 to 188)	1 131	15.1 (13.7 to 16.6)		150 (135 to 165)	1 104	13.6 (12.2 to 14.9)		−22.5 (−33.1 to −9.8)	−10.2 (−21.9 to 2.9)	−30.4 (−40.0 to −19.3)
Eswatini	88 (74 to 102)	573	15.4 (13.0 to 17.8)		97 (83 to 111)	637	15.2 (13.1 to 17.4)		97 (81 to 113)	683	14.2 (11.8 to 16.6)		−0.8 (−19.4 to 23.0)	−6.8 (−25.5 to 15.4)	−7.6 (−26.9 to 15.5)
Ethiopia	3 481 (3 147 to 3 815)	35 455	9.8 (8.9 to 10.8)		4 139 (3 753 to 4 525)	48 262	8.6 (7.8 to 9.4)		5020 (4 521 to 5 519)	60 379	8.3 (7.5 to 9.1)		−12.7 (−23.7 to −0.1)	−3.1 (−15.7 to 10.8)	−15.3 (−26.3 to −2.6)
Fiji	140 (121 to 159)	527	26.6 (22.9 to 30.3)		124 (104 to 144)	610	20.3 (17.1 to 23.6)		123 (104 to 142)	613	20.1 (16.9 to 23.3)		−23.6 (−38.9 to −5.5)	−1.2 (−21.6 to 24.5)	−24.5 (−38.9 to −6.9)
Finland	1 037 (974 to 1 100)	4 247	24.4 (22.9 to 25.9)		1 091 (1 030 to 1 152)	4 479	24.4 (23.0 to 25.7)		1 063 (1 000 to 1 126)	4 600	23.1 (21.7 to 24.5)		−0.3 (−8.4 to 8.7)	−5.1 (−12.7 to 3.1)	−5.4 (−13.3 to 3.0)
France	12 448 (11 977 to 12 919)	47 857	26.0 (25.0 to 27.0)		14 400 (13 876 to 14 924)	51 261	28.1 (27.1 to 29.1)		15 780 (15 127 to 16 433)	52 869	29.9 (28.6 to 31.1)		8.0 (2.4 to 13.8)	6.3 (0.5 to 12.3)	14.8 (8.4 to 21.5)
Gabon	106 (90 to 122)	725	14.6 (12.4 to 16.9)		98 (83 to 113)	1 016	9.6 (8.1 to 11.2)		102 (86 to 118)	1 273	8.0 (6.7 to 9.3)		−34.1 (−47.3 to −17.2)	−16.9 (−34.5 to 4.5)	−45.2 (−56.3 to −31.3)
Gambia	130 (111 to 149)	697	18.6 (15.9 to 21.4)		151 (130 to 172)	984	15.4 (13.2 to 17.5)		167 (142 to 192)	1 194	14.0 (11.9 to 16.1)		−17.6 (−32.6 to 1.3)	−8.9 (−25.8 to 11.2)	−25.0 (−39.4 to −7.0)
Georgia	1 168 (1 018 to 1 318)	3 454	33.8 (29.5 to 38.2)		1331 (1 194 to 1 468)	3 363	39.6 (35.5 to 43.6)		1 328 (1 195 to 1 461)	3 242	41.0 (36.9 to 45.1)		17.0 (−0.6 to 38.6)	3.5 (−10.5 to 19.1)	21.1 (3.2 to 43.2)
Germany	21 570 (20 612 to 22 528)	68 643	31.4 (30.0 to 32.8)		22 884 (21 999 to 23 769)	69 860	32.8 (31.5 to 34.0)		23 580 (22 639 to 24 521)	71 249	33.1 (31.8 to 34.4)		4.2 (−1.6 to 10.6)	1.0 (−4.5 to 6.8)	5.3 (−0.8 to 11.9)
Ghana	1 453 (1 294 to 1 612)	11 095	13.1 (11.7 to 14.5)		2 105 (1 886 to 2 324)	15 045	14.0 (12.5 to 15.5)		2 343 (2087 to 2 599)	17 652	13.3 (11.8 to 14.7)		6.8 (−8.2 to 24.5)	−5.1 (−18.5 to 10.6)	1.4 (−13.4 to 18.6)
Greece	2 384 (2 235 to 2 533)	9 411	25.3 (23.8 to 26.9)		2 331 (2 206 to 2 456)	9 249	25.2 (23.9 to 26.6)		2 287 (2 166 to 2 408)	9 091	25.2 (23.8 to 26.5)		−0.5 (−8.2 to 8.2)	−0.2 (−7.5 to 7.5)	−0.7 (−8.5 to 8.0)
Grenada	7 (4 to 10)	68	10.2 (5.6 to 14.8)		5 (3 to 7)	81	6.2 (3.3 to 9.1)		5 (3 to 7)	85	5.9 (3.5 to 8.3)		−39.4 (−70.8 to 11.0)	−4.6 (−53.7 to 103.8)	−42.2 (−71.2 to 4.8)
Guatemala	594 (542 to 646)	6 556	9.1 (8.3 to 9.9)		689 (629 to 749)	8 871	7.8 (7.1 to 8.4)		812 (739 to 885)	10 670	7.6 (6.9 to 8.3)		−14.3 (−24.1 to −3.0)	−2.0 (−13.5 to 10.4)	−16.0 (−25.8 to −4.8)
Guinea	735 (648 to 822)	4 414	16.7 (14.7 to 18.6)		964 (855 to 1 073)	5 486	17.6 (15.6 to 19.6)		1 088 (959 to 1 217)	6 498	16.7 (14.8 to 18.7)		5.5 (−10.7 to 25.0)	−4.7 (−19.7 to 12.1)	0.6 (−14.9 to 19.0)
Guinea–Bissau	114 (99 to 129)	656	17.4 (15.1 to 19.7)		116 (100 to 132)	866	13.4 (11.5 to 15.3)		120 (101 to 139)	1 023	11.7 (9.9 to 13.6)		−22.9 (−36.6 to −6.6)	−12.5 (−29.8 to 8.1)	−32.5 (−45.2 to −17.4)
Guyana	99 (86 to 112)	480	20.6 (17.9 to 23.4)		110 (95 to 125)	508	21.7 (18.7 to 24.6)		124 (106 to 142)	548	22.6 (19.4 to 25.9)		5.1 (−13.7 to 26.8)	4.5 (−14.1 to 27.2)	9.8 (−10.4 to 34.0)
Haiti	1 320 (1 149 to 1 491)	5 057	26.1 (22.7 to 29.5)		1 232 (1 087 to 1 377)	6 341	19.4 (17.2 to 21.7)		1 702 (1 479 to 1 925)	7 154	23.8 (20.7 to 26.9)		−25.6 (−37.8 to −11.3)	22.5 (2.7 to 45.8)	−8.9 (−24.4 to 9.3)
Honduras	349 (300 to 398)	3 759	9.3 (8.0 to 10.6)		451 (394 to 508)	5 192	8.7 (7.6 to 9.8)		596 (521 to 671)	6 211	9.6 (8.4 to 10.8)		−6.4 (−22.4 to 12.8)	10.5 (−7.7 to 32.5)	3.4 (−14.5 to 24.9)
Hungary	1 789 (1 674 to 1904)	8 501	21.1 (19.7 to 22.4)		1724 (1 635 to 1 813)	8 452	20.4 (19.4 to 21.4)		1 789 (1 701 to 1 877)	8 351	21.4 (20.4 to 22.5)		−3.1 (−10.7 to 5.4)	5.0 (−2.1 to 12.8)	1.8 (−6.0 to 10.5)
Iceland	49 (42 to 56)	215	22.8 (19.7 to 25.8)		63 (56 to 70)	254	24.8 (22.0 to 27.7)		57 (50 to 64)	265	21.5 (18.7 to 24.3)		9.1 (−7.4 to 29.0)	−13.5 (−27.5 to 3.1)	−5.6 (−21.1 to 12.7)
India	259 538 (239 324 to 279 752)	689 671	37.6 (34.7 to 40.6)		297 751 (272 846 to 322 656)	853 996	34.9 (32.0 to 37.8)		341 389 (310 810 to 371 968)	954 548	35.8 (32.6 to 39.0)		−7.4 (−17.4 to 3.9)	2.6 (−9.5 to 15.8)	−5.0 (−16.0 to 7.0)
Indonesia	53 737 (48 354 to 59 120)	146 595	36.7 (33.0 to 40.3)		70 309 (63 435 to 77 183)	172 118	40.9 (36.9 to 44.8)		81 643 (73 154 to 90 132)	190 328	42.9 (38.4 to 47.4)		11.4 (−3.3 to 28.8)	5.0 (−9.1 to 20.9)	17.0 (1.0 to 35.5)
Iran (Islamic Republic of)	8 881 (7 692 to 10 070)	43 336	20.5 (17.8 to 23.2)		9 714 (8 553 to 10 875)	56 027	17.3 (15.3 to 19.4)		9 174 (8 066 to 10 282)	60 442	15.2 (13.4 to 17.0)		−15.4 (−29.3 to 1.4)	−12.5 (−26.3 to 4.1)	−25.9 (−38.3 to −11.2)
Iraq	2 477 (2 139 to 2 815)	13 410	18.5 (16.0 to 21.0)		2 692 (2 336 to 3 048)	17 308	15.6 (13.5 to 17.6)		2 969 (2 564 to 3 374)	22 295	13.3 (11.5 to 15.1)		−15.8 (−30.5 to 2.4)	−14.4 (−29.6 to 3.7)	−27.9 (−40.4 to −12.4)
Ireland	916 (850 to 982)	2 971	30.8 (28.6 to 33.1)		703 (657 to 749)	3 608	19.5 (18.2 to 20.8)		768 (719 to 817)	3 674	20.9 (19.6 to 22.3)		−36.8 (−42.8 to −30.2)	7.3 (−2.2 to 17.6)	−32.2 (−38.4 to −25.1)
Israel	580 (541 to 619)	4 276	13.6 (12.7 to 14.5)		623 (588 to 658)	5 344	11.7 (11.0 to 12.3)		656 (618 to 694)	5 844	11.2 (10.6 to 11.9)		−14.0 (−21.2 to −6.2)	−3.7 (−11.2 to 4.4)	−17.2 (−24.2 to −9.5)
Italy	17 306 (16 572 to 18 040)	48 571	35.6 (34.1 to 37.1)		19 218 (18 456 to 19 980)	50 973	37.7 (36.2 to 39.2)		19 285 (18 445 to 20 125)	52 410	36.8 (35.2 to 38.4)		5.8 (−0.2 to 12.1)	−2.4 (−8.1 to 3.4)	3.3 (−2.9 to 9.8)
Jamaica	343 (310 to 376)	1 801	19 (17.2 to 20.9)		288 (259 to 317)	2 051	14.0 (12.6 to 15.5)		311 (280 to 342)	2 200	14.1 (12.7 to 15.6)		−26.3 (−36.0 to −15.3)	0.7 (−12.9 to 16.2)	−25.8 (−35.5 to −14.6)
Japan	28 463 (26 669 to 30 257)	108 671	26.2 (24.5 to 27.8)		35 259 (33 578 to 36 940)	111 376	31.7 (30.2 to 33.2)		36 687 (35 064 to 38 310)	111 264	33 (31.5 to 34.4)		20.9 (11.5 to 30.9)	4.2 (−2.4 to 11.0)	25.9 (16.7 to 36.0)
Jordan	517 (446 to 588)	3 093	16.7 (14.4 to 19.0)		653 (562 to 744)	4 540	14.4 (12.4 to 16.4)		864 (742 to 986)	6 166	14.0 (12.0 to 16.0)		−13.9 (−29.3 to 5.0)	−2.6 (−20.2 to 18.8)	−16.2 (−31.2 to 1.8)
Kazakhstan	3 912 (3 582 to 4 242)	10 815	36.2 (33.1 to 39.2)		3 581 (3 281 to 3 881)	12 343	29.0 (26.6 to 31.4)		3 131 (2 864 to 3 398)	12 962	24.2 (22.1 to 26.2)		−19.8 (−28.8 to −9.7)	−16.7 (−26.2 to −6.2)	−33.2 (−40.8 to −24.4)
Kenya	815 (735 to 895)	17 501	4.7 (4.2 to 5.1)		909 (824 to 994)	23 772	3.8 (3.5 to 4.2)		1 184 (1 063 to 1 305)	28 988	4.1 (3.7 to 4.5)		−17.9 (−28 to −6.0)	6.8 (−7.0 to 22.8)	−12.3 (−24.0 to 0.9)
Kiribati	3 (1 to 5)	51	5.9 (1.9 to 9.9)		5 (3 to 7)	66	7.6 (4.2 to 11.0)		9 (6 to 12)	73	12.3 (8.9 to 15.6)		28.4 (−43.8 to 331.9)	61.6 (−2.7 to 213.2)	107.5 (9.2 to 532.5)
Kuwait	134 (109 to 159)	1 465	9.2 (7.4 to 10.9)		265 (222 to 308)	2 297	11.5 (9.7 to 13.4)		421 (348 to 494)	3 114	13.5 (11.2 to 15.9)		26.1 (−1.9 to 63.6)	17.2 (−8.0 to 49.1)	47.8 (14.7 to 93.2)
Kyrgyzstan	924 (845 to 1 003)	3 201	28.9 (26.4 to 31.3)		871 (789 to 953)	3 801	22.9 (20.8 to 25.1)		823 (738 to 908)	4 135	19.9 (17.8 to 22.0)		−20.6 (−30.2 to −9.7)	−13.2 (−24.6 to −0.3)	−31.1 (−40.0 to −21.3)
Lao People's Democratic Republic	1 084 (982 to 1 186)	3 015	36.0 (32.6 to 39.3)		1 276 (1 155 to 1 397)	3 972	32.1 (29.1 to 35.2)		1 364 (1 232 to 1 496)	4 573	29.8 (26.9 to 32.7)		−10.7 (−21.9 to 2.1)	−7.1 (−19.0 to 6.2)	−17.0 (−27.5 to −4.5)
Latvia	668 (592 to 744)	1 958	34.1 (30.2 to 38)		499 (445 to 553)	1 820	27.4 (24.5 to 30.4)		374 (336 to 412)	1 674	22.4 (20.1 to 24.6)		−19.6 (−31.2 to −5.9)	−18.5 (−29.4 to −5.4)	−34.5 (−43.7 to −23.5)
Lebanon	597 (491 to 703)	2 648	22.6 (18.5 to 26.6)		763 (624 to 902)	3674	20.8 (17.0 to 24.5)		1 026 (842 to 1 210)	4 902	20.9 (17.2 to 24.7)		−7.9 (−29.1 to 18.8)	0.8 (−22.2 to 29.9)	−7.2 (−28.4 to 19.6)
Lesotho	254 (223 to 285)	1 219	20.8 (18.3 to 23.4)		278 (245 to 311)	1 299	21.4 (18.8 to 24.0)		278 (242 to 314)	1 386	20.1 (17.5 to 22.7)		2.7 (−13.3 to 22.3)	−6.2 (−21.6 to 11.7)	−3.7 (−19.6 to 15.3)
Liberia	269 (232 to 306)	1 632	16.5 (14.2 to 18.8)		369 (319 to 419)	2 217	16.7 (14.4 to 18.9)		366 (313 to 419)	2 669	13.7 (11.7 to 15.7)		1.0 (−17.2 to 22.8)	−17.6 (−32.7 to 0.3)	−16.8 (−32.5 to 2.0)
Libya	673 (580 to 766)	3544	19.0 (16.4 to 21.6)		749 (650 to 848)	4 416	17.0 (14.7 to 19.2)		821 (712 to 930)	4 627	17.7 (15.4 to 20.1)		−10.7 (−26.3 to 8.4)	4.6 (−13.5 to 26.1)	−6.6 (−22.8 to 13.7)
Lithuania	737 (667 to 807)	2 800	26.3 (23.8 to 28.8)		671 (603 to 739)	2 663	25.2 (22.6 to 27.8)		528 (472 to 584)	2 466	21.4 (19.1 to 23.7)		−4.3 (−17.0 to 10.2)	−15.1 (−26.6 to −1.7)	−18.7 (−29.5 to −6.0)
Luxembourg	87 (78 to 96)	353	24.6 (22.0 to 27.2)		94 (85 to 103)	418	22.5 (20.4 to 24.6)		96 (86 to 106)	485	19.8 (17.6 to 22.0)		−8.7 (−21.0 to 5.9)	−12.0 (−24.1 to 1.5)	−19.6 (−31.4 to −6.0)
Madagascar	2 435 (2 214 to 2 656)	8 648	28.2 (25.6 to 30.7)		2 692 (2 457 to 2 927)	11 946	22.5 (20.6 to 24.5)		2 771 (2 524 to 3 018)	14 614	19.0 (17.3 to 20.7)		−20.0 (−29.4 to −9.2)	−15.9 (−25.6 to −4.6)	−32.7 (−40.8 to −23.6)
Malawi	744 (652 to 836)	6 011	12.4 (10.8 to 13.9)		588 (514 to 662)	7 800	7.5 (6.6 to 8.5)		621 (541 to 701)	9 517	6.5 (5.7 to 7.4)		−39.1 (−49.2 to −27.5)	−13.4 (−27.7 to 3.7)	−47.3 (−56.0 to −36.9)
Malaysia	3 036 (2 735 to 3 337)	15 458	19.6 (17.7 to 21.6)		3 682 (3 285 to 4 079)	20 321	18.1 (16.2 to 20.1)		4 176 (3 714 to 4 638)	23 101	18.1 (16.1 to 20.1)		−7.8 (−20.5 to 6.9)	−0.2 (−14.6 to 16.7)	−8.0 (−20.9 to 7.0)
Maldives	43 (36 to 50)	166	25.9 (21.9 to 29.8)		37 (30 to 44)	273	13.5 (11.1 to 16.0)		36 (29 to 43)	377	9.6 (7.9 to 11.3)		−47.6 (−59.4 to −33.6)	−29.5 (−45.5 to −8.3)	−63.1 (−71.0 to −53.4)
Mali	1 081 (962 to 1 200)	5 849	18.5 (16.5 to 20.5)		1 115 (1 007 to 1 223)	7 903	14.1 (12.7 to 15.5)		1 279 (1 143 to 1 415)	9 368	13.7 (12.2 to 15.1)		−23.7 (−34.1 to −11.6)	−3.2 (−16.5 to 11.2)	−26.1 (−36.7 to −13.7)
Malta	66 (58 to 74)	315	20.9 (18.4 to 23.5)		75 (67 to 83)	352	21.3 (18.9 to 23.7)		95 (85 to 105)	374	25.4 (22.6 to 28.2)		1.8 (−13.8 to 20.7)	19.2 (1.8 to 39.9)	21.4 (2.9 to 42.9)
Mauritania	187 (163 to 211)	1 485	12.6 (11.0 to 14.2)		237 (207 to 267)	2 053	11.5 (10.1 to 13.0)		291 (254 to 328)	2 481	11.7 (10.3 to 13.2)		−8.3 (−23.7 to 9.9)	1.6 (−15.3 to 21.8)	−6.9 (−22.4 to 11.7)
Mauritius	169 (149 to 189)	880	19.2 (17.0 to 21.5)		136 (121 to 151)	974	14.0 (12.4 to 15.5)		170 (151 to 189)	1 023	16.6 (14.8 to 18.4)		−27.3 (−38.4 to −14.1)	19.0 (2.2 to 39.3)	−13.5 (−26.4 to 1.9)
Mexico	8 331 (7 713 to 8 949)	65 080	12.8 (11.9 to 13.8)		11 054 (10 215 to 11 893)	80 427	13.7 (12.7 to 14.8)		12 249 (11 229 to 13 269)	89 657	13.7 (12.5 to 14.8)		7.4 (−3.3 to 19.8)	−0.6 (−11.3 to 11.1)	6.7 (−4.6 to 19.5)
Micronesia (Federated States of)	2 (0 to 5)	64	3.1 (0.0 to 7.3)		6 (3 to 9)	66	9.1 (5.2 to 13.0)		5 (2 to 8)	75	6.7 (4.4 to 9.0)		190.4 (2.0 to 1 498.4)	−26.1 (−61.1 to 37.9)	114.8 (−39.2 to 1 199.2)
Mongolia	545 (483 to 607)	1564	34.9 (30.9 to 38.8)		580 (506 to 654)	1 985	29.2 (25.5 to 33.0)		630 (540 to 720)	2 157	29.2 (25.0 to 33.4)		−16.2 (−29.4 to −0.7)	0.0 (−17.7 to 21.4)	−16.2 (−30.2 to 0.5)
Montenegro	84 (72 to 96)	482	17.4 (15.0 to 19.9)		80 (69 to 91)	504	15.9 (13.7 to 18.1)		73 (62 to 84)	512	14.3 (12.1 to 16.4)		−9.0 (−25.3 to 11.1)	−10.2 (−27.1 to 10.5)	−18.2 (−33.4 to 0.8)
Morocco	4 923 (4 273 to 5 573)	19 151	25.7 (22.3 to 29.1)		3 743 (3 261 to 4 225)	23 121	16.2 (14.1 to 18.3)		3 869 (3 370 to 4 368)	25 416	15.2 (13.3 to 17.2)		−37.0 (−47.6 to −24.0)	−6.0 (−21.5 to 13.1)	−40.8 (−50.9 to −28.8)
Mozambique	1 491 (1 301 to 1 681)	9829	15.2 (13.2 to 17.1)		1 218 (1 073 to 1 363)	12 770	9.5 (8.4 to 10.7)		1 192 (1 046 to 1 338)	15 247	7.8 (6.9 to 8.8)		−37.1 (−47.3 to −24.7)	−18.0 (−31.5 to −2.9)	−48.5 (−56.8 to −38.3)
Myanmar	9 656 (8 760 to 10 552)	31 530	30.6 (27.8 to 33.5)		12 397 (11 326 to 13 468)	35 397	35.0 (32.0 to 38.1)		13 995 (12 723 to 15 267)	38 548	36.3 (33.0 to 39.6)		14.4 (0.9 to 30.3)	3.7 (−8.7 to 17.7)	18.6 (4.3 to 34.8)
Namibia	177 (155 to 199)	1 040	17.0 (14.9 to 19.2)		173 (151 to 195)	1 326	13.1 (11.4 to 14.7)		180 (155 to 205)	1 489	12.1 (10.4 to 13.8)		−23.3 (−35.9 to −8.6)	−7.3 (−23.8 to 12.0)	−28.9 (−41.6 to −14.3)
Nepal	6 113 (5 610 to 6 616)	14 134	43.3 (39.7 to 46.8)		7 396 (6 722 to 8 070)	17 210	43.0 (39.1 to 46.9)		8 041 (7 285 to 8 797)	18 457	43.6 (39.5 to 47.7)		−0.6 (−12.2 to 12.0)	1.4 (−11.1 to 15.9)	0.7 (−11.1 to 13.8)
Netherlands (Kingdom of the)	5 460 (5 208 to 5 712)	12 985	42.1 (40.1 to 44.0)		5 397 (5 158 to 5 636)	13 760	39.2 (37.5 to 41.0)		5 530 (5 264 to 5 796)	14 166	39.0 (37.2 to 40.9)		−6.7 (−12.4 to −0.5)	−0.5 (−7.0 to 6.1)	−7.2 (−13.1 to −1.0)
New Zealand	986 (924 to 1 048)	2 982	33.1 (31.0 to 35.1)		999 (946 to 1 052)	3 474	28.8 (27.2 to 30.3)		965 (911 to 1 019)	3 736	25.8 (24.4 to 27.3)		−13.0 (−19.8 to −5.5)	−10.2 (−17.1 to −3.0)	−21.9 (−28.1 to −14.9)
Nicaragua	312 (274 to 350)	3 059	10.2 (9.0 to 11.5)		330 (292 to 368)	3 884	8.5 (7.5 to 9.5)		382 (338 to 426)	4 356	8.8 (7.8 to 9.8)		−16.7 (−29.5 to −1.1)	3.2 (−12.2 to 22.0)	−14.0 (−27.6 to 2.3)
Niger	820 (735 to 905)	5 861	14.0 (12.5 to 15.4)		1 259 (1 132 to 1 386)	8 226	15.3 (13.8 to 16.9)		1476 (1 321 to 1 631)	10 362	14.2 (12.8 to 15.7)		9.4 (−5.1 to 26.4)	−6.9 (−19.7 to 7.6)	1.8 (−11.9 to 18.4)
Nigeria	8484 (7 686 to 9 282)	68 963	12.3 (11.2 to 13.5)		10 142 (9 189 to 11 095)	88 706	11.4 (10.4 to 12.5)		12 472 (11 217 to 13 727)	103 941	12.0 (10.8 to 13.2)		−7.1 (−18.5 to 5.9)	5.0 (−8.5 to 20.7)	−2.5 (−15.0 to 11.8)
North Macedonia	262 (228 to 296)	1573	16.7 (14.5 to 18.8)		263 (232 to 294)	1 700	15.5 (13.7 to 17.3)		262 (233 to 291)	1 732	15.1 (13.4 to 16.8)		−7.1 (−22.1 to 11.0)	−2.2 (−16.8 to 14.9)	−9.1 (−23.4 to 8.8)
Norway	900 (849 to 951)	3 600	25.0 (23.6 to 26.4)		1 027 (975 to 1 079)	3 967	25.9 (24.6 to 27.2)		985 (930 to 1 040)	4 316	22.8 (21.5 to 24.1)		3.6 (−4.2 to 11.8)	−11.9 (−18.2 to −4.9)	−8.7 (−15.7 to −1.2)
Oman	192 (163 to 221)	1 426	13.5 (11.4 to 15.5)		215 (181 to 249)	2 260	9.5 (8.0 to 11.0)		275 (235 to 315)	3 500	7.9 (6.7 to 9.0)		−29.3 (−43.5 to −11.7)	−17.4 (−33.4 to 3.0)	−41.6 (−52.6 to −28.1)
Pakistan	26 540 (23 610 to 29 470)	82 611	32.1 (28.6 to 35.7)		32 087 (28 492 to 35 682)	111 791	28.7 (25.5 to 31.9)		36 167 (32 021 to 40 313)	130 939	27.6 (24.5 to 30.8)		−10.7 (−23.7 to 5.1)	−3.8 (−18.4 to 13.5)	−14.0 (−26.7 to 0.8)
Panama	234 (207 to 261)	2 061	11.4 (10.1 to 12.7)		297 (265 to 329)	2 582	11.5 (10.3 to 12.7)		315 (281 to 349)	2 923	10.8 (9.6 to 11.9)		1.3 (−13.3 to 18.7)	−6.3 (−19.6 to 9.2)	−5.1 (−18.4 to 11.3)
Papua New Guinea	741 (660 to 822)	3 523	21.0 (18.7 to 23.3)		879 (784 to 974)	4 510	19.5 (17.4 to 21.6)		1 146 (1 016 to 1 276)	5 256	21.8 (19.3 to 24.3)		−7.4 (−20.5 to 7.5)	11.9 (−4.7 to 31.0)	3.7 (−11.8 to 21.7)
Paraguay	629 (559 to 699)	3 275	19.2 (17.1 to 21.4)		684 (611 to 757)	4 190	16.3 (14.6 to 18.1)		756 (673 to 839)	4 743	15.9 (14.2 to 17.7)		−15.0 (−27.4 to −0.6)	−2.4 (−16.5 to 14.0)	−17.0 (−29.4 to −2.8)
Peru	2 912 (2 652 to 3 172)	17 347	16.8 (15.3 to 18.3)		3 201 (2 907 to 3 495)	20 292	15.8 (14.3 to 17.2)		3 465 (3 127 to 3 803)	22 545	15.4 (13.9 to 16.9)		−6.0 (−17.4 to 6.6)	−2.6 (−14.9 to 11.6)	−8.4 (−19.7 to 4.7)
Philippines	10 903 (10 027 to 11 779)	47 988	22.7 (20.9 to 24.6)		20 071 (18 327 to 21 815)	62 026	32.4 (29.6 to 35.2)		24 260 (22 038 to 26 482)	70 573	34.4 (31.2 to 37.5)		42.4 (26.1 to 60.2)	6.2 (−6.6 to 20.5)	51.3 (34.1 to 70.8)
Poland	6 542 (6 051 to 7 033)	31 016	21.1 (19.5 to 22.7)		7 895 (7 462 to 8 328)	32 497	24.3 (23 to 25.6)		8 625 (8 174 to 9 076)	32 372	26.6 (25.3 to 28.0)		15.2 (5.3 to 26.8)	9.7 (1.6 to 18.4)	26.3 (15.4 to 39.0)
Portugal	2 317 (2 124 to 2 510)	8 646	26.8 (24.6 to 29.0)		1 941 (1 825 to 2057)	9 010	21.5 (20.3 to 22.8)		1 722 (1 626 to 1 818)	8 893	19.4 (18.3 to 20.4)		−19.6 (−27.3 to −10.7)	−10.1 (−17.0 to −2.6)	−27.7 (−34.4 to −19.9)
Qatar	23 (18 to 28)	440	5.2 (4.0 to 6.4)		53 (43 to 63)	1 617	3.3 (2.7 to 3.9)		98 (80 to 116)	2 296	4.3 (3.5 to 5.1)		−37.3 (−53.3 to −13.1)	30.2 (−0.5 to 70.4)	−18.3 (−39.3 to 12.5)
Republic of Korea	6 562 (5 986 to 7 138)	37 612	17.5 (15.9 to 19.0)		5963 (5 581 to 6 345)	41 568	14.4 (13.4 to 15.3)		6 110 (5 749 to 6 471)	44 102	13.9 (13.0 to 14.7)		−17.8 (−26.3 to −8.3)	−3.4 (−11.5 to 5.3)	−20.6 (−28.4 to −11.4)
Republic of Moldova	739 (651 to 827)	3 217	23.0 (20.3 to 25.7)		688 (608 to 768)	3 411	20.2 (17.8 to 22.5)		530 (462 to 598)	3 424	15.5 (13.5 to 17.5)		−12.2 (−25.6 to 3.7)	−23.3 (−35.5 to −8.8)	−32.6 (−43.5 to −19.6)
Romania	6 220 (5 637 to 6 803)	18 023	34.5 (31.3 to 37.8)		5 040 (4 605 to 5 475)	17 242	29.2 (26.7 to 31.8)		4 328 (3 988 to 4 668)	16 743	25.9 (23.8 to 27.9)		−15.3 (−25.4 to −3.7)	−11.6 (−21.2 to −0.5)	−25.1 (−33.7 to −15.4)
Russian Federation	38 866 (35 118 to 42 614)	119 694	32.5 (29.3 to 35.6)		30 272 (27 460 to 33 084)	122 062	24.8 (22.5 to 27.1)		25 039 (22 857 to 27 221)	120 156	20.8 (19.0 to 22.7)		−23.6 (−33.4 to −12.3)	−16.0 (−25.9 to −4.6)	−35.8 (−43.7 to −27.0)
Rwanda	656 (586 to 726)	4 413	14.9 (13.3 to 16.4)		388 (345 to 431)	5 869	6.6 (5.9 to 7.3)		436 (382 to 490)	6 964	6.3 (5.5 to 7.0)		−55.5 (−61.8 to −48.0)	−5.3 (−19.7 to 12.0)	−57.9 (−64.2 to −50.4)
Saint Lucia	15 (11 to 19)	106	14.1 (10.0 to 18.2)		11 (7 to 15)	134	8.2 (5.5 to 11.0)		14 (10 to 18)	145	9.6 (7.7 to 11.6)		−41.8 (−60.2 to −15.9)	17.2 (−16.6 to 69.7)	−31.8 (−51.4 to −3.1)
Saint Vincent and the Grenadines	2 (0 to 5)	74	2.7 (0.0 to 6.5)		6 (4 to 8)	80	7.5 (4.5 to 10.4)		8 (5 to 11)	84	9.5 (6.6 to 12.5)		175.9 (−485.7 to 1 503.7)	27.6 (−23.9 to 125.0)	252.1 (5.9 to 2 245.7)
Samoa	20 (15 to 25)	103	19.4 (14.7 to 24.0)		16 (12 to 20)	115	14.0 (10.4 to 17.5)		13 (9 to 17)	119	10.9 (7.9 to 13.9)		−27.9 (−51.3 to 5.5)	−21.9 (−48.9 to 16.5)	−43.7 (−62.6 to −17.1)
Sao Tome and Principe	5 (2 to 8)	79	6.3 (2.8 to 9.9)		3 (0 to 6)	102	2.9 (0.3 to 5.6)		3 (0 to 6)	116	2.6 (1.1 to 4.1)		−53.5 (−85.7 to 8.6)	−11.7 (−74.7 to 181.6)	−58.9 (−87.7 to −6.5)
Saudi Arabia	1 881 (1 610 to 2 152)	12 755	14.8 (12.6 to 16.9)		2 511 (2 144 to 2 878)	19 276	13.0 (11.1 to 14.9)		3 128 (2 622 to 3 634)	24 162	13.0 (10.9 to 15.0)		−11.7 (−27.6 to 8.6)	−0.6 (−20.7 to 24.4)	−12.2 (−30.0 to 9.0)
Senegal	681 (607 to 755)	5 417	12.6 (11.2 to 13.9)		820 (734 to 906)	7 162	11.5 (10.3 to 12.7)		817 (731 to 903)	8 490	9.6 (8.6 to 10.6)		−8.9 (−21.8 to 6.1)	−16.0 (−27.5 to −2.3)	−23.5 (−34.3 to −11.0)
Serbia	2 305 (2 085 to 2 525)	7 543	30.6 (27.6 to 33.5)		1 999 (1 839 to 2 159)	7 433	26.9 (24.7 to 29.1)		1 769 (1 642 to 1 896)	7 439	23.8 (22.1 to 25.5)		−12.0 (−22.2 to 0.1)	−11.6 (−20.7 to −1.2)	−22.2 (−31.0 to −12.3)
Seychelles	0 (0 to 2)	58	0.0 (0.0 to 3.6)		0 (0 to 3)	70	0.0 (0.0 to 3.6)		4 (1 to 7)	73	5.5 (3.5 to 7.4)		Incalculable	Incalculable	Incalculable!
Sierra Leone	1 061 (931 to 1 191)	2 560	41.5 (36.4 to 46.5)		1 097 (965 to 1 229)	3 654	30.0 (26.4 to 33.6)		1 127 (983 to 1 271)	4 277	26.4 (23.0 to 29.7)		−27.6 (−39.0 to −13.9)	−12.2 (−26.5 to 4.9)	−36.4 (−46.6 to −24.2)
Singapore	677 (611 to 743)	3 275	20.7 (18.7 to 22.7)		701 (629 to 773)	4 413	15.9 (14.3 to 17.5)		741 (661 to 821)	4 959	14.9 (13.3 to 16.6)		−23.2 (−33.6 to −11.4)	−5.9 (−19.0 to 9.5)	−27.7 (−37.5 to −16.5)
Slovakia	466 (430 to 502)	4 335	10.8 (9.9 to 11.6)		436 (406 to 466)	4 576	9.5 (8.9 to 10.2)		433 (404 to 462)	4 611	9.4 (8.8 to 10.0)		−11.4 (−20.3 to −1.8)	−1.5 (−10.6 to 8.3)	−12.7 (−21.1 to −3.6)
Slovenia	459 (428 to 490)	1 674	27.4 (25.5 to 29.3)		482 (452 to 512)	1 757	27.4 (25.7 to 29.2)		478 (447 to 509)	1 768	27.0 (25.3 to 28.8)		0.0 (−8.7 to 9.6)	−1.4 (−10.2 to 8.2)	−1.4 (−10.3 to 8.4)
Solomon Islands	52 (44 to 60)	240	21.7 (18.3 to 25.1)		57 (48 to 66)	313	18.2 (15.5 to 21.0)		62 (53 to 71)	369	16.8 (14.3 to 19.3)		−16.0 (−33.0 to 4.8)	−7.8 (−26.2 to 14.9)	−22.6 (−37.6 to −3.2)
Somalia	946 (848 to 1 044)	4 685	20.2 (18.1 to 22.3)		1 051 (943 to 1 159)	6 242	16.8 (15.1 to 18.6)		1 292 (1 152 to 1 432)	7 526	17.2 (15.3 to 19.0)		−16.6 (−27.9 to −3.7)	2.0 (−12.1 to 18.3)	−15.0 (−27.2 to −1.4)
South Africa	5 842 (5 368 to 6 316)	29 740	19.6 (18.1 to 21.2)		5 972 (5 476 to 6 468)	36 020	16.6 (15.2 to 18.0)		6 121 (5 545 to 6 697)	39 787	15.4 (13.9 to 16.8)		−15.6 (−24.9 to −5.2)	−7.2 (−18.3 to 5.1)	−21.7 (−30.9 to −11.2)
South Sudan	582 (518 to 646)	3 421	17.0 (15.1 to 18.9)		686 (612 to 760)	5 370	12.8 (11.4 to 14.2)		764 (676 to 852)	62 53	12.2 (10.8 to 13.6)		−24.9 (−35.6 to −12.3)	−4.4 (−18.7 to 12.2)	−28.2 (−38.7 to −15.8)
Spain	8 083 (7 755 to 8 411)	34 808	23.2 (22.3 to 24.2)		8 720 (8 388 to 9 052)	39 988	21.8 (21.0 to 22.6)		8 783 (8 417 to 9 149)	39 749	22.1 (21.2 to 23.0)		−6.1 (−11.2 to −0.7)	1.3 (−4.3 to 7.3)	−4.8 (−10.2 to 0.9)
Sri Lanka	3374 (3 060 to 3 688)	13 754	24.5 (22.3 to 26.8)		3 520 (3 191 to 3 849)	15 120	23.3 (21.1 to 25.5)		4 074 (3 678 to 4 470)	15 851	25.7 (23.2 to 28.2)		−5.1 (−16.9 to 8.1)	10.4 (−3.7 to 26.5)	4.8 (−8.5 to 19.2)
Sudan	4 315 (3 802 to 4 828)	15 328	28.2 (24.8 to 31.5)		4854 (4 260 to 5 448)	19 693	24.7 (21.6 to 27.7)		5 531 (4 824 to 6 238)	23 435	23.6 (20.6 to 26.6)		−12.4 (−26.2 to 4.1)	−4.3 (−19.9 to 14.4)	−16.2 (−29.6 to −0.4)
Suriname	72 (60 to 84)	317	22.7 (19.1 to 26.4)		66 (54 to 78)	374	17.7 (14.6 to 20.8)		74 (61 to 87)	409	18.1 (15.1 to 21.1)		−22.2 (−38.7 to −1.3)	2.5 (−19.6 to 30.4)	−20.3 (−36.8 to 0.6)
Sweden	1 787 (1 690 to 1 884)	7 245	24.7 (23.3 to 26.0)		1 804 (1 717 to 1 891)	7 840	23.0 (21.9 to 24.1)		1 808 (1 715 to 1901)	8 126	22.3 (21.1 to 23.4)		−6.7 (−13.4 to 0.3)	−3.3 (−10.0 to 3.7)	−9.8 (−16.4 to −2.9)
Switzerland	1 742 (1 655 to 1 829)	5 898	29.5 (28.1 to 31.0)		1 786 (1 699 to 1 873)	6 633	26.9 (25.6 to 28.2)		1 779 (1 685 to 1 873)	7 137	24.9 (23.6 to 26.2)		−8.8 (−15.0 to −2.2)	−7.4 (−13.9 to −0.6)	−15.6 (−21.5 to −9.4)
Syrian Arab Republic	1 717 (1 499 to 1935)	9 680	17.7 (15.5 to 20.0)		2 319 (2033 to 2 605)	13 375	17.3 (15.2 to 19.5)		2 299 (2 010 to 2 588)	11 813	19.5 (17.0 to 21.9)		−2.3 (−18.2 to 17.4)	12.3 (−6.3 to 34.7)	9.7 (−7.9 to 31.4)
Tajikistan	517 (457 to 577)	3 573	14.5 (12.8 to 16.2)		636 (562 to 710)	4 842	13.1 (11.6 to 14.7)		751 (662 to 840)	5 533	13.6 (12.0 to 15.2)		−9.2 (−23.1 to 6.9)	3.3 (−12.9 to 22.2)	−6.2 (−20.4 to 10.6)
Thailand	12 638 (11 837 to 13 439)	47 856	26.4 (24.7 to 28.1)		14 079 (13 190 to 14 968)	54 304	25.9 (24.3 to 27.6)		14 753 (13 820 to 15 686)	56 801	26.0 (24.3 to 27.6)		−1.8 (−10.2 to 7.3)	0.2 (−8.6 to 9.5)	−1.7 (−10.0 to 7.9)
Timor-Leste	100 (87 to 113)	488	20.5 (17.8 to 23.2)		120 (105 to 135)	628	19.1 (16.8 to 21.4)		126 (110 to 142)	745	16.9 (14.7 to 19.1)		−6.9 (−22.3 to 11.6)	−11.5 (−26.1 to 5.4)	−17.6 (−31.7 to −0.6)
Togo	678 (596 to 760)	2 795	24.3 (21.3 to 27.2)		792 (700 to 884)	3674	21.6 (19.1 to 24.1)		852 (745 to 959)	4 363	19.5 (17.1 to 22.0)		−11.2 (−24.4 to 5.0)	−9.4 (−23.8 to 7.4)	−19.5 (−32.8 to −4.3)
Tonga	5 (2 to 8)	60	8.3 (4.1 to 12.5)		4 (2 to 6)	65	6.2 (2.4 to 9.9)		5 (2 to 8)	65	7.7 (4.3 to 11.2)		−25.9 (−68.6 to 60.5)	25.9 (−41.6 to 199.2)	−6.7 (−55.7 to 93.8)
Trinidad and Tobago	199 (174 to 224)	943	21.1 (18.5 to 23.8)		203 (178 to 228)	1 053	19.3 (16.9 to 21.6)		203 (178 to 228)	1 093	18.6 (16.3 to 20.8)		−8.7 (−23.2 to 8.6)	−3.7 (−19.0 to 14.2)	−12.0 (−26.1 to 4.7)
Tunisia	1 868 (1 601 to 2 135)	6 839	27.3 (23.4 to 31.2)		2 084 (1 810 to 2 358)	8 154	25.6 (22.2 to 28.9)		2 132 (1 852 to 2 412)	8 591	24.8 (21.6 to 28.1)		−6.4 (−23.2 to 14.4)	−2.9 (−19.6 to 17.5)	−9.1 (−25.1 to 10.5)
Türkiye	15 326 (13 926 to 16 726)	43 887	34.9 (31.7 to 38.1)		18 341 (16 875 to 19 807)	52 870	34.7 (31.9 to 37.5)		18 923 (17 388 to 20 458)	59 612	31.7 (29.2 to 34.3)		−0.7 (−11.9 to 12.4)	−8.5 (−18.5 to 2.9)	−9.1 (−19.6 to 3.0)
Turkmenistan	455 (397 to 513)	2 878	15.8 (13.8 to 17.8)		470 (408 to 532)	3 586	13.1 (11.4 to 14.8)		567 (488 to 646)	3 917	14.5 (12.5 to 16.5)		−17.1 (−31.2 to −0.6)	10.4 (−9.1 to 33.4)	−8.4 (−24.4 to 9.9)
Uganda	1 090 (978 to 1 202)	11 906	9.2 (8.2 to 10.1)		1 311 (1 185 to 1 437)	16 508	7.9 (7.2 to 8.7)		1 595 (1 435 to 1 755)	20 747	7.7 (6.9 to 8.5)		−13.3 (−24.6 to −0.1)	−3.2 (−16.0 to 10.9)	−16.0 (−27.1 to −3.2)
Ukraine	17 716 (15 976 to 19 456)	40 478	43.8 (39.5 to 48.1)		14 024 (12 458 to 15 590)	39 323	35.7 (31.7 to 39.6)		11 756 (10 499 to 13 013)	37 812	31.1 (27.8 to 34.4)		−18.5 (−29.9 to −5.4)	−12.8 (−25.4 to 1.8)	−29.0 (−38.7 to −17.9)
United Arab Emirates	226 (194 to 258)	2318	9.8 (8.4 to 11.1)		472 (402 to 542)	7425	6.4 (5.4 to 7.3)		658 (555 to 761)	8 011	8.2 (6.9 to 9.5)		−34.8 (−46.8 to −19.8)	29.2 (3.9 to 60.2)	−15.8 (−32.0 to 4.3)
United Kingdom	20 730 (19 817 to 21 643)	47 709	43.5 (41.5 to 45.4)		21 721 (20 971 to 22 471)	52 357	41.5 (40.1 to 42.9)		22 532 (21 700 to 23 364)	54 636	41.2 (39.7 to 42.8)		−4.5 (−9.8 to 1.0)	−0.6 (−5.4 to 4.6)	−5.1 (−10.3 to 0.5)
United Republic of Tanzania	1 937 (1 748 to 2 126)	18 502	10.5 (9.5 to 11.5)		2 343 (2 116 to 2 570)	24 446	9.6 (8.7 to 10.5)		2 862 (2 565 to 3 159)	29 438	9.7 (8.7 to 10.7)		−8.5 (−20.2 to 5.5)	1.4 (−12.0 to 17.0)	−7.1 (−19.6 to 7.2)
United States	62 672 (60 425 to 64 919)	220 572	28.4 (27.4 to 29.4)		59 334 (57 407 to 61 261)	246 576	24.1 (23.3 to 24.8)		61 229 (59 102 to 63 356)	261 567	23.4 (22.6 to 24.2)		−15.3 (−19.3 to −11.1)	−2.7 (−7.2 to 2.0)	−17.6 (−21.7 to −13.5)
Uruguay	729 (669 to 789)	2 505	29.1 (26.7 to 31.5)		683 (634 to 732)	2 613	26.1 (24.3 to 28.0)		646 (601 to 691)	2 707	23.9 (22.2 to 25.5)		−10.2 (−19.4 to −0.2)	−8.7 (−17.4 to 1.0)	−18.0 (−26.2 to −8.6)
Uzbekistan	2 411 (2 144 to 2 678)	15 538	15.5 (13.8 to 17.2)		2 293 (1997 to 2 589)	20 208	11.4 (9.9 to 12.8)		2 467 (2 138 to 2 796)	22 516	11 (9.5 to 12.4)		−26.9 (−38.4 to −13.6)	−3.4 (−19.6 to 16.6)	−29.4 (−40.8 to −16.2)
Vanuatu	15 (11 to 19)	108	13.9 (9.8 to 18.0)		19 (14 to 24)	146	13.0 (9.7 to 16.3)		22 (17 to 27)	170	12.9 (9.9 to 15.9)		−6.1 (−34.5 to 35.0)	−0.6 (−29.7 to 40.9)	−6.7 (−34.0 to 33.1)
Venezuela (Bolivarian Republic of)	2 581 (2 308 to 2 854)	15 973	16.2 (14.5 to 17.9)		3 000 (2 685 to 3 315)	19 934	15.1 (13.5 to 16.6)		3 328 (2 974 to 3 682)	21 428	15.5 (13.9 to 17.2)		−6.9 (−19.7 to 8.6)	3.2 (−11.2 to 20.1)	−3.9 (−17.3 to 11.8)
Viet Nam	12 575 (11 388 to 13 762)	54 680	23.0 (20.8 to 25.2)		15 616 (14 218 to 17 014)	67 183	23.2 (21.2 to 25.3)		17 618 (15 979 to 19 257)	72 068	24.5 (22.2 to 26.7)		1.1 (−11.0 to 15.4)	5.2 (−7.7 to 19.8)	6.3 (−7.2 to 21.9)
Yemen	3 048 (2 677 to 3 419)	8 891	34.3 (30.1 to 38.5)		3 709 (3 246 to 4 172)	13 274	27.9 (24.5 to 31.4)		4 204 (3 653 to 4 755)	16 202	26.0 (22.5 to 29.4)		−18.5 (−31.7 to −2.7)	−7.1 (−22.5 to 10.8)	−24.3 (−36.7 to −9.7)
Zambia	615 (550 to 680)	5 582	11.0 (9.9 to 12.2)		656 (589 to 723)	7 172	9.2 (8.2 to 10.1)		683 (609 to 757)	8 863	7.7 (6.9 to 8.5)		−17.0 (−28.5 to −3.3)	−15.8 (−27.5 to −2.3)	−30.1 (−40.0 to −18.7)
Zimbabwe	1 469 (1 320 to 1 618)	6 884	21.3 (19.2 to 23.5)		1 316 (1 194 to 1 438)	7 414	17.8 (16.1 to 19.4)		1 148 (1 032 to 1 264)	8 044	14.3 (12.8 to 15.7)		−16.8 (−27.4 to −4.0)	−19.6 (−30.1 to −7.9)	−33.1 (−42.0 to −22.8)

UR: uncertainty range; WHO: World Health Organization.

^a^ Source: WHO and International Labour Organization (ILO) joint estimates of the work-related burden of disease and injury.^10^

^b^ Source: United Nations population estimates.^26^

Note: The WHO/ILO joint estimates^10^ are produced for the 183 countries for which WHO produces global health estimates on burden of disease.^17^ These countries have populations greater than 90 000 people.

Data sources: WHO and International Labour Organization joint estimates of the work-related burden of disease and injury,.^10^^–^^13^^. ^and United Nations population estimates.^26^

### By disease group

**Fig. 3:**
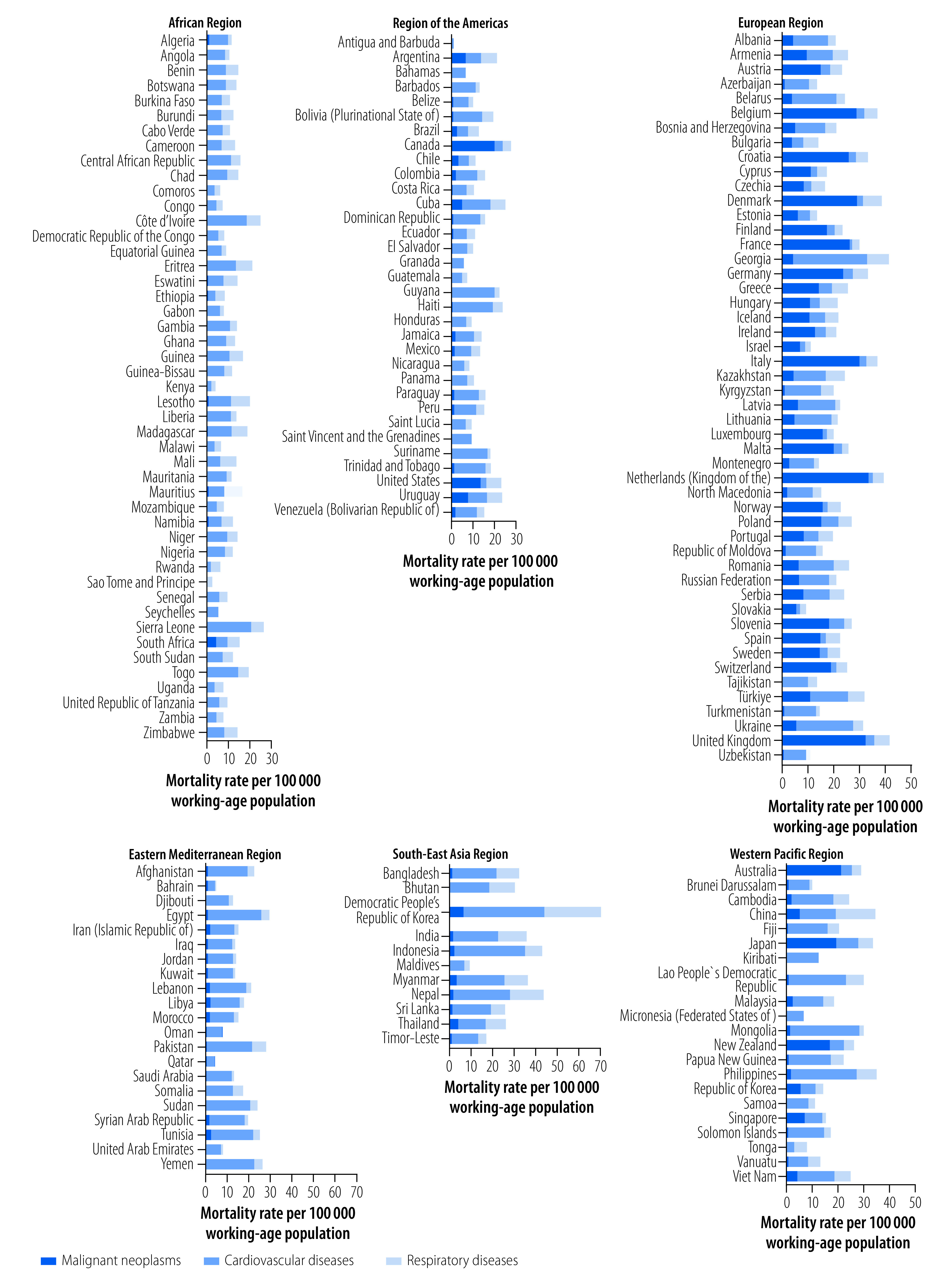
Mortality rate per 100 000 working-age population (≥ 15 years) from diseases attributable to selected occupational risk factors, by World Health Organization region and disease group in 183 countries, 2016

Of the three disease groups, cardiovascular diseases accounted for the highest global work-related mortality rate in 2016: 13.6 deaths per 100 000 working-age population (95% UR: 12.9 to 14.3).^28^ The corresponding mortality rates were 5.3 deaths per 100 000 (95% UR: 5.2 to 5.4) for malignant neoplasms, and 8.8 deaths per 100 000 (95% UR: 8.4 to 9.1) for respiratory diseases. For the African, Eastern Mediterranean, South-East Asia and Western Pacific Regions, the cardiovascular diseases group also contributed most to the mortality rate (Fig. 3). In contrast, work-related malignant neoplasms contributed most to the mortality rates in the Region of the Americas and European Region. 

### By occupational risk factor

The pattern of mortality rates by occupational risk factor corresponded to that by disease group, since these are linked in exposure–disease pairs (Table 1).^28^ Exposure to long working hours contributed the largest proportion of deaths from work-related diseases (corresponding to cardiovascular diseases). Among the regions, long working hours accounted for the largest proportion of deaths in the African, Eastern Mediterranean, South-East Asia and Western Pacific Regions. However, in the Region of the Americas and European Region, occupational exposure to carcinogens (corresponding to malignant neoplasms) contributed the largest proportion of deaths. 

### By sex

Males had a higher global mortality rate attributed to work-related diseases (40.1 deaths per 100 000 working-age population; 95% UR: 38.6 to 41.5) than did females (15.3 deaths per 100 000; 95% UR: 14.6 to 16.0).^28^ Mortality rates for work-related diseases were higher for males than females in all regions.

### By age group

Global mortality rates from work-related diseases were higher in older people than in younger people, reflecting general patterns of noncommunicable disease.^29^ People aged 85–89 years had the highest global mortality rate from work-related diseases (246.9 deaths per 100 000 population; 95% UR: 228.0 to 265.9), whereas the youngest age group of 15–19 years had the lowest rate (0.1 deaths per 100 000; 95% UR: 0.1 to 0.1).^28^ When plotting the mortality rate by 5-year age groups (combining those ≥ 65 years due to small numbers), the highest rates were in the African Region for the two youngest age groups and the South-East Asia Region for all other age groups. The lowest mortality rates occurred in the European and Western Pacific Regions, up to age 24–29 years, above which the Region of the Americas had the lowest age-specific rates.^28^

### Trends

Between 2000 and 2016, the global mortality rate from selected work-related diseases decreased from 30.7 to 27.7 per 100 000 population, a percentage change of −9.8 (95% UR: −13.3 to −6.1; Table 2). This change was driven by the mortality rate dropping between 2000 and 2010 (−9.1%; 95% UR: −12.4 to −5.6), whereas between 2010 and 2016 there was only a small decrease (−0.8%; 95% UR: −4.7 to 3.2). Overall, between 2000 and 2016, the mortality rate decreased in all regions, except South-East Asia (1.6%; 95% UR: −6.6 to 10.3). The largest decrease was observed for the Region of the Americas and the Eastern Mediterranean Region (−16.9%; 95% UR: −19.4 to −14.2, and −15.2%; 95% UR: −21.2 to −9.0, respectively). Each region’s contribution to the global mortality rate varied over time with its population share of the total mortality rate.

## Policy implications

### National monitoring

In 2008–2009, national information systems for workers’ health existed in 51 (42%) out of 121 of countries.^30^ In Table 3 we present case studies from four countries across different regions, describing their current monitoring system for workers’ health and showing how the new indicator could be integrated into national information systems.^31^^–^^41^ We also suggest potential facilitators and barriers to the integration of the new indicator into national monitoring systems, and its predicted impact. The case studies suggest that the new indicator would fill a gap in current monitoring systems, and could serve as an addition rather than a replacement for existing indicators. Feedback from country respondents demonstrated that none of their monitoring systems include the new indicator (Table 3). Some countries already had similar indicators, calculated using national data sources and using various methods that limit international comparability of their indicators. For other countries, the indicator could not be calculated from currently available data.

**Table 3:** Country case studies of how the new global indicator for work-related burden of disease can be integrated into official national monitoring systems for workers’ health

Country (Region)	National monitoring systems for workers’ health	Similar indicators in the national monitoring systems	How the new indicator could be integrated into national monitoring systems	Potential facilitators and barriers for use of the new indicator	Predicted impact of the new indicator
China (Western Pacific Region)	The national workers’ health monitoring system of China comprises the national surveillance system of occupational disease^31^ and the national surveillance system for occupational hazards in the workplace.^32^^,^^33^ The agency responsible for the former monitoring system is the Chinese Center for Disease Control and Prevention of the National Health Commission. The occupational disease surveillance system is the most relevant to the new indicator. It is a network-based reporting system that includes all relevant agencies in China. The system reports new cases of occupational disease covering a total of 121 diseases in nine categories, and relevant variables such as age, job title, duration of work and industrial sector.	The relevant indicators in the national monitoring system are: (i) number of workers exposed to occupational risk factors (data collected in national statistical surveys); (ii) number of diagnosed cases of occupational disease (data collected by the Agency of Occupational Disease Diagnosis based on clinical diagnosis and the occupational exposure experience); and (iii) number of deaths due to occupational disease (data collected from the national mortality surveillance system). The monitoring system does not include the new indicator.	The new indicator could be included in the national monitoring system without replacing any current indicators. The new indicator could be calculated using number of deaths due to occupational disease as the numerator, obtained from national data sources. However, the national workers’ health monitoring system can only collect the approximate number of deaths due to occupational disease, so only an approximate of the new indicator can be calculated.	*Facilitators* The new indicator can be used to better evaluate the burden of disease caused by occupational risk factors in China. *Barriers* The variables needed to calculate the indicator from national data sources are still not accurate enough. The country therefore needs to develop a better evaluation method. The cause of death data for occupational diseases are currently not classified according to the ICD-10. China is working on linking the country’s classification of occupational disease to ICD-10.	The new indicator can better describe the situation of occupational health status in China. The indicator would provide solid evidence for policy-makers to allocate occupational disease prevention resources. The new indicator can accelerate integration of the surveillance system of occupational disease with the national mortality surveillance system. The new indicator can help to deepen collaboration among different departments within the health system. The indicator can help to integrate data across different systems in China: the surveillance system of occupational disease and health hazards, the national surveillance system for occupational hazards in the workplace, the national mortality surveillance system and the population-based cancer registration system.
Islamic Republic of Iran (Eastern Mediterranean Region)	Workers’ health in Islamic Republic of Iran is monitored through occupational health examinations in accordance with the laws of the country. The statistics of these examinations are uploaded to the portal of the Deputy Minister of Health. Access to this portal is only available to the health ministry, which supervises occupational health examinations.	The relevant indicators in the national monitoring system are: (i) coverage of occupational health examinations (percentage of employees for whom occupational health examinations were performed); (ii) percentage of employees with work-related diseases (this indicator is available for a limited number of occupational diseases); and (iii) percentage of the workforce who are exposed to risk factors in workplaces. These indicators are calculated from the data of the registration system of occupational health examinations and occupational health inspection. The monitoring system does not include the new indicator.	The new indicator could be included in the national monitoring system of workers’ health promotional indicators without replacing any current indicators. Some necessary components for calculating the new indicator from national data sources are unavailable to the health ministry. There is no occupational health examination registration system for recording occupational health examinations for each person, and as a result, electronic occupational health records do not exist for each employee.	*Facilitators* The new indicator is relevant to the Islamic Republic of Iran. The country has limited indicators in the field of occupational diseases. Indicators related to burden of disease are not calculated. *Barriers* The country lacks a system or platform for gathering data to calculate the new indicator. The required data are not available to the health ministry.	The Islamic Republic of Iran can use the new indicator to supplement the existing information on workers’ health, and to inform future policies and plans to control occupational diseases. The new indicator can help to improve the system of providing occupational health services to employees. The indicator could strengthen the health system by including the data of diseases attributable to selected occupational risk factors, by disease, risk factor, sex and age group in the current surveillance and disease control systems. The new indicator would be effective for decision-making by other organizations. It could help departments to carry out interventions and workforce planning, and increase the productivity of employees. Gathering mortality rate data would require communication and synergy among different departments of the health ministry and other organizations.
Italy (European Region)	The Italian national monitoring system for occupational diseases comprises three main systems that capture different occupational diseases’ characteristics and incidence: (i) ReNaM, the Italian national mesothelioma registry;^34^^a^ (ii) MalProf, an epidemiological surveillance system for occupational diseases;^35^^b^ and (iii) the INAIL database,^36^ the database of compensation claims and reported cases collected through the public insurance for workers against occupational injuries and diseases.^c^ The agency responsible for these monitoring systems is the Italian National Institute for Insurance against Accidents at Work. The INAIL database is the most relevant to the new indicator and comprises three indicators: (i) total number of claims for occupational diseases; (ii) total number of compensated cases of occupational diseases; and (iii) total number of cases of fatal occupational diseases.	The relevant indicator in the national monitoring system is: total number of compensated cases of fatal occupational diseases (INAIL database). The monitoring system does not include the new indicator.	The new indicator could be added to the monitoring system without replacing any current indicators. The new indicator could be calculated using the number of compensated cases of fatal occupational diseases from national data sources as the numerator. Italy’s national monitoring system includes data and indicators on 19 of the 21 exposure–disease pairs captured by the new indicator. It may be possible to compare the value of the indicator when generated using different data sources (that is, estimated deaths from WHO/ILO joint estimates^d^ versus reported cases from national data collections).	*Facilitators* The new indicator could promote awareness about emerging occupational diseases, improving the efficiency of the health insurance system in Italy. The new indicator could promote systematic comparisons between insurance data and data from epidemiological surveillance systems. *Barriers* Regions collect the data on reported fatal cases of occupational disease (required for the new indicator) at regional level. They are required to collect and provide these data, but their operating capacity to do this differs. The centralized agency that oversees the three national monitoring systems could potentially harmonize these regional data sets to produce the numerator for the indicator.	The new indicator may allow a comparison of estimates of the numbers of deaths due to work-related diseases with the numbers of deaths reported through the current monitoring systems in Italy. The new indicator could contribute to expanding the list of occupational diseases by adding newly recognized causal links with specific occupations. The new indicator could improve knowledge and awareness about the etiology of occupational diseases to inform the occupational safety and public health of communities. The new indicator could increase Italy’s capacity to estimate the occupational fraction of its burden of disease.
South Africa (African Region)	Occupational diseases are monitored in the national occupational mortality surveillance South Africa.^37^ The agency responsible for this system is the National Institute for Occupational Health. The data do not permit perfect attribution of fatal cases of disease to occupational risk factors. The system comprises 14 indicators, but is still in development.	The relevant indicators in the national occupational mortality surveillance are: (i) occupational exposure to asbestos (mining), mesothelioma (ii) occupational exposure to silica, silicosis; and (iii) occupational asthmagens (all occupations), asthma. These indicators are calculated as proportional mortality ratios. These ratios estimate risk of death from the disease in a specific occupation, compared to in the general population. Their calculation requires mortality data by occupation. South Africa’s official statistics agency provides such data up to the year 2016, but does not code mortality data for more recent years by occupation. Additionally, indicators on selected occupational risk factors are available from national surveys conducted by the national statistics agency: for example on exposure to long working hours from the national income dynamics study.^38^ The monitoring system does not include the new indicator.	The new indicator could be added to the monitoring system without replacing any current indicators. The new indicator cannot be calculated from current national data sources. National data on fatal cases of occupational diseases (the numerator) are limited in availability and quality. National data on the total population (the denominator) are available annually from estimates of the national statistics agency.^39^	*Facilitators* Adding the new indicator to the national occupational mortality surveillance system will provide important occupational health surveillance and assist with recognition of diseases as work-related in South Africa. There is open-access availability to the new indicator via the WHO/ILO joint estimates.^d^ *Barriers* Data access and linkages across departments would be a barrier to generating the new indicator from national data sources. The responsible agency would need to adjust any data received. Caution should be taken when interpreting the new indicator due to the quality of the data and variability in people’s access to health care.	Adding the new indicator to the national monitoring system can support the evolving surveillance of disease mortality and attribution where it is currently limited. The indicator can broaden the scope of workers’ health monitoring in South Africa. The new indicator can support policy development and implementation on occupational health. The new indicator can increase awareness in the health sector about the importance of occupational risk factors, and build capacity and stimulate research on this topic. The indicator has the potential to inform integration of basic occupational health services into the primary health-care system. The new indicator could foster linkages across the departments of health, labour and home affairs^40^^,^^41^ to enable them to handle occupational health monitoring jointly.

ICD-10: International Statistical Classification of Diseases and Related Health Problems, 10th revision; ILO: International Labour Organization; WHO: World Health Organization.

^a^ ReNaM is the Italian national mesothelioma registry, an epidemiological surveillance system focused on active search of mesothelioma incident cases. The registry provides a wide range of information on mesothelioma cases, including the retrospective analysis of data on exposure to asbestos collected via questionnaire on occupational and medical history.

^b^ MalProf is an epidemiological surveillance system for occupational diseases based on the observations of Local Health Units through their Prevention Services, by collating information from judicial claims and from an active search of cases. The system allows identification of causal link between the disease and the employment condition.

^c^ The INAIL database is the database of the Italian National Institute for Insurance against Accidents at Work and occupational diseases. The database collates results from the activities of the public insurance system for workers against injuries at work and occupational diseases, and includes both compensation claims and defined cases.

^d^ WHO/ILO joint estimates of the work-related burden of disease and injury.^10^^–^^13^

Country respondents suggested potential benefits of the indicator that could facilitate its introduction into monitoring systems (Table 3). Benefits include providing additional information to evaluate the occupational disease burden, and generate greater awareness of emerging occupational diseases. The relevance and ready availability of the indicator in the WHO/ILO joint estimates is also a facilitator to its use. Barriers to use of the indicator are the need for countries to generate the indicator themselves by collecting, linking and analysing the necessary input data.

Several potential positive impacts of the new indicator were mentioned (Table 3). Country respondents suggested that the indicator could improve the monitoring of workers’ health and strengthen the attribution of occupational and work-related diseases, thereby potentially improving coverage of workers’ compensation and therefore the basic level of the social protection floor. The indicator could also promote awareness of occupational risk factors and work-related diseases among health policy-makers, and foster action within the health and other sectors.

Next steps for countries could include adoption of the indicator into their national monitoring systems and promotion of the indicator among their networks, including national, regional and global authorities responsible for workers’ health and sustainable development monitoring. Until countries are able to collect their own data for the number of work-related deaths, they can use the WHO/ILO joint estimates, as presented in this article. Current estimates were published in September 2021,^10^ but the indicator can be produced annually, using updates to the WHO estimates of the total number of deaths per disease^25^ and population-attributable fractions for occupational risk factors.^10^^–^^13^ The indicator is also available disaggregated by sex and age group for monitoring health inequalities among workers,^42^ ensuring that no one is left behind in sustainable development.^1^

### Global and regional monitoring

In Fig. 1 we show how the indicator is already being integrated into global and regional monitoring systems. At the global level, WHO is using the indicator in its environmental health monitoring system,^43^ and making it available open access via the WHO Occupational Burden of Disease Application.^19^At the regional level, the European Commission has pledged to collaborate to establish the new indicator and include it in the global indicator framework for the SDGs:

“Cooperation with the ILO and WHO on data and knowledge […] will include support, together with Member States, for the creation of a new indicator on mortality from diseases attributed to occupational risk factors as part of the United Nations sustainable development goals.” (p. 20).^44^

Eurostat, the European Union’s statistical office, has produced and published experimental statistics on the number of incident cases of selected occupational diseases for Member States.^45^

### Strengths and weaknesses

The new indicator has several strengths. First, using the United Nations established methods^9^ and open-access data sources,^10^^,^^26^ the indicator captures the great majority of the total estimated work-related deaths.^10^^–^^13^ Second, as the number of work-related deaths (the numerator) is estimated based on modelling of exposure data and relative risks, these estimates are less at risk of misreporting bias than estimates based on reported or compensated cases of fatal occupational or work-related disease. Third, the indicator can also be used to monitor other health topics, such as the social and commercial determinants of health (as a proxy for exposure to unhealthy working conditions), and noncommunicable diseases (as the fraction of these diseases that can be addressed by health protection and promotion in workplaces). Finally, WHO has already produced this indicator (shown here for 183 countries), disaggregated by sex and age group, allowing epidemiological analysis and health inequality monitoring,^42^^,^^46^ without any additional reporting burden for countries.

The indicator also has limitations. First, it captures deaths only from exposure–disease pairs for which there is sufficient evidence, and not for all such pairings. The recent addition of two new exposure–disease pairings (deaths from ischaemic heart disease or from stroke attributable to long working hours)^12^ added almost 40% of the total estimated number of deaths attributable to occupational risk factors (744 924 out of 1 879 890 deaths in 2016).^13^ Other exposure–disease pairs (if any) must be added to the comparative risk assessment when sufficient evidence is available to support this. Additional pairings could include occupational exposure to ultraviolet radiation and non-melanoma skin cancer,^47^ and occupational exposure to welding fumes and trachea, bronchus and lung cancer.^48^ Second, the scope of the work-related burden of disease does not include mortality among child labourers; mortality due to secondary exposures or take-home of exposures to families or other community members; or intergenerational mortality due to occupational risk factors. Third, the indicator could be further disaggregated by migrant status of workers to align with the full definition of SDG target 8.8.1 and by whether the worker works in the informal or formal economy, but this would require disaggregated input data, which are currently sparse.^49^^,^^50^ Finally, the quality of the indicator depends on the input data. Governments are encouraged to continue their work to provide large-scale, high-quality, official data on individuals’ exposure to occupational risk factors and on causes of death and disease.

### Alternative specification

There are possible alternative specifications for the indicator. The data source for the numerator could be reported fatal or incident cases of occupational disease, or national occupational burden of diseases estimates, as already monitored in some countries (Table 3). Nevertheless, the advantage of using the WHO/ILO joint estimates is that they produce comparable estimates for 183 countries and the six WHO regions and their population cohorts. Alternatively, using disability-adjusted life-years attributable to occupational risk factors would capture both mortality and morbidity.

The use of total population as the denominator would facilitate comparisons with other mortality rate indicators that use the total population, such as those in the global indicator framework for the SDGs.^9^ However, as the data for the numerator are collected for people aged 15 years and older, we believe that the working-age population is the most appropriate denominator for the indicator.

## Conclusions

The workplace is an important setting for preventing disease.^51^ A key first step when improving working conditions is understanding and quantifying the risk factors and their attributable burdens. With the current focus of monitoring on occupational injuries, the majority of work-related mortality is not captured. To assess progress towards health targets for workers, international organizations, regions and countries must expand their indicators to consider mortality from work-related diseases. Integration of this new indicator in global, regional and national monitoring systems will improve the comprehensiveness, accuracy and harmonization of workers’ health and sustainable development surveillance. The indicator will provide opportunities for analysing health inequalities among workers, both within and between countries. The indicator can provide an improved evidence base for developing effective health policy and systems for workers, and can play a key role in assessing progress in countries’ policy commitments to workers’ health.^1^^–^^5^

## References

[1] Resolution A/RES/70/1. Transforming our world: the 2030 agenda for sustainable development. In: Seventieth United Nations General Assembly, New York, 25 September 2015. New York: United Nations; 2015. Available from: http://www.un.org/ga/search/view_doc.asp?symbol=A/RES/70/1&Lang=E [cited 2023 Apr 19].

[2] WHO global strategy on health, environment and climate change: the transformation needed to improve lives and well-being sustainably through healthy environments. Geneva: World Health Organization; 2020. Available from: https://www.who.int/publications/i/item/9789240000377 [cited 2023 Apr 19].

[3] Plan of action on workers’ health 2015–2025. Washington, DC: Pan American Health Organization; 2017. Available from: https://iris.paho.org/handle/10665.2/33986 [cited 2023 Apr 19].

[4] International labour standards on occupational safety and health. Geneva: International Labour Organization; 2022. Available from: https://www.ilo.org/global/standards/subjects-covered-by-international-labour-standards/occupational-safety-and-health/lang — en/index.htm [cited 2022 Aug 11].

[5] Resolution on the inclusion of a safe and healthy working environment in the ILO’s framework of fundamental principles and rights at work [ILC.110/Resolution I]. In: 110th International Labour Conference, Geneva, 27 May to 11 June 2022. Geneva: International Labour Organization; 2022. Available from: https://www.ilo.org/ilc/ILCSessions/110/reports/texts-adopted/WCMS_848632/lang--en/index.htm [cited 2023 Apr 19].

[6] Closing the gap in a generation: health equity through action on the social determinants of health. Final Report of the Commission on Social Determinants of Health. Geneva: World Health Organization; 2008. Available from: https://apps.who.int/iris/handle/10665/43943 [cited 2023 Apr 19].

[7] Kickbusch I. Health in all policies. BMJ. 2013 Jul 3;347 jul03 1:f4283. 10.1136/bmj.f4283 23824002

[8] SDG indicator metadata: Indicator 8.8.1: fatal and non-fatal occupational injuries per 100 000 workers, by sex and migrant status. New York: United Nations; 2022. Available from: https://unstats.un.org/sdgs/metadata/files/Metadata-08-08-01.pdf [cited 2023 Apr 19].

[9] Global indicator framework for the sustainable development goals and targets of the 2030 agenda for sustainable development. New York: United Nations; 2022. Available from: https://unstats.un.org/sdgs/indicators/indicators-list/ [cited 2023 Apr 19].

[10] WHO/ILO Joint Estimates of the Work-related Burden of Disease and Injury. 2000-2016: global monitoring report. Geneva: World Health Organization; 2021. Available from: https://www.who.int/publications/i/item/9789240034945 [cited 2023 Apr 19].

[11] WHO/ILO Joint Estimates of the Work-related Burden of Disease and Injury. 2000-2016: technical report with data sources and methods. Geneva: World Health Organization; 2021. Available from: https://www.who.int/publications/i/item/9789240034921 [cited 2023 Apr 19].

[12] Pega F, Náfrádi B, Momen NC, Ujita Y, Streicher KN, Prüss-Üstün AM, et al. Technical Advisory Group. Global, regional, and national burdens of ischemic heart disease and stroke attributable to exposure to long working hours for 194 countries, 2000-2016: a systematic analysis from the WHO/ILO joint estimates of the work-related burden of disease and injury. Environ Int. 2021 Sep;154:106595. 10.1016/j.envint.2021.106595 34011457 PMC8204267

[13] Pega F, Hamzaoui H, Náfrádi B, Momen NC. Global, regional and national burden of disease attributable to 19 selected occupational risk factors for 183 countries, 2000-2016: a systematic analysis from the WHO/ILO joint estimates of the work-related burden of disease and injury. Scand J Work Environ Health. 2022 Mar 1;48(2):158–68. 10.5271/sjweh.4001 34806754 PMC9045235

[14] WHA71.2. Thirteenth general programme of work, 2019–2023. In: Seventy-first World Health Assembly, Geneva, 21–26 May 2018. Geneva: World Health Organization; 2018. Available from: https://apps.who.int/gb/e/e_wha71.html [cited 2023 Apr 19].

[15] The thirteenth general programme of work 2019–2023: promote health, keep the world safe, serve the vulnerable. Geneva: World Health Organization; 2019. Available from: https://www.who.int/about/what-we-do/thirteenth-general-programme-of-work-2019 — 2023 [cited 2023 Apr 19].

[16] Stanaway JD, Afshin A, Gakidou E, Lim SS, Abate D, Abate KH, et al. GBD 2017 Risk Factor Collaborators. Global, regional, and national comparative risk assessment of 84 behavioural, environmental and occupational, and metabolic risks or clusters of risks for 195 countries and territories, 1990-2017: a systematic analysis for the Global Burden of Disease Study 2017. Lancet. 2018 Nov 10;392(10159):1923–94. 10.1016/S0140-6736(18)32225-6 30496105 PMC6227755

[17] WHO methods and data sources for global burden of disease estimates 2000–2019. Global health estimates technical paper WHO/DDI/DNA/GHE/2020.3. Geneva: World Health Organization; 2020. Available from: https://www.who.int/docs/default-source/gho-documents/global-health-estimates/ghe2019_cod_methods.pdf [cited 2023 Apr 19].

[18] Stevens GA, Alkema L, Black RE, Boerma JT, Collins GS, Ezzati M, et al. The GATHER Working Group. Guidelines for accurate and transparent health estimates reporting: the GATHER statement. Lancet. 2016 Dec 10;388(10062):e19–23. 10.1016/S0140-6736(16)30388-9 27371184

[19] Occupational burden of disease application: app for exploring and visualizing occupational burden of disease estimates, by country, sex and age group. Version 1.0 [internet]. Geneva: World Health Organization; 2021. Available from: https://who-ilo-joint-estimates.shinyapps.io/OccupationalBurdenOfDisease/ [cited 2023 Apr 19].

[20] Ezzati M, Lopez AD, Rodgers A, Murray CJL, editors. Comparative quantification of health risks: global and regional burden of disease attributable to selected major risk factors. Geneva: World Health Organization; 2004.

[21] Náfrádi B, Kiiver H, Neupane S, Momen NC, Streicher KN, Pega F. Estimating the population exposed to a risk factor over a time window: a microsimulation modelling approach from the WHO/ILO joint estimates of the work-related burden of disease and injury. PLoS One. 2022 Dec 30;17(12):e0278507. 10.1371/journal.pone.0278507 36584100 PMC9803131

[22] Descatha A, Sembajwe G, Pega F, Ujita Y, Baer M, Boccuni F, et al. The effect of exposure to long working hours on stroke: a systematic review and meta-analysis from the WHO/ILO joint estimates of the work-related burden of disease and injury. Environ Int. 2020 Sep;142:105746. 10.1016/j.envint.2020.105746 32505015

[23] Li J, Pega F, Ujita Y, Brisson C, Clays E, Descatha A, et al. The effect of exposure to long working hours on ischaemic heart disease: a systematic review and meta-analysis from the WHO/ILO joint estimates of the work-related burden of disease and injury. Environ Int. 2020 Sep;142:105739. 10.1016/j.envint.2020.105739 32505014 PMC7339147

[24] Pega F, Momen NC, Ujita Y, Driscoll T, Whaley P. Systematic reviews and meta-analyses for the WHO/ILO joint estimates of the work-related burden of disease and injury. Environ Int. 2021 Oct;155:106605. 10.1016/j.envint.2021.106605 34051644 PMC8287588

[25] Global health estimates 2016 [internet]. Geneva: World Health Organization; 2018. Available from: https://www.who.int/gho/mortality_burden_disease/en/ [cited 2018 May 11].

[26] World population prospects 2019 [online]. New York: United Nations; 2019. Available from: https://population.un.org/wpp/ [cited 2023 Apr 19].

[27] Efron B. Bootstrap methods: another look at the jackknife. Ann Stat. 1979;7(1):1–26. 10.1214/aos/1176344552

[28] Pega F, Al-Emam R, Cao B, Davis CW, Edwards SJ, Gagliardi D, et al. New global indicator for workers’ health: mortality rate from diseases attributable to selected occupational risk factors: supplementary files [online repository]. London: figshare; 2023. 10.6084/m9.figshare.22661311 PMC10225940 37265682

[29] Bennett JE, Stevens GA, Mathers CD, Bonita R, Rehm J, Kruk ME, et al. NCD Countdown 2030 collaborators. NCD Countdown 2030: worldwide trends in noncommunicable disease mortality and progress towards sustainable development goal target 3.4. Lancet. 2018 Sep 22;392(10152):1072–88. 10.1016/S0140-6736(18)31992-5 30264707

[30] WHO global plan of action on workers’ health (2008–2017): baseline for implementation. Global country survey 2008/2009. Executive summary and survey findings. Geneva: World Health Organization; 2013. Available from: https://www.who.int/publications/i/item/WHO-FWC-PHE-2013-01 [cited 2023 Apr 19].

[31] Zheng J, Zhang S, Wang H, Yu Y, Hu W. Surveillance of noise exposure level in the manufacturing industry – China, 2020. China CDC Wkly. 2021 Oct 22;3(43):906–10. 34745689 10.46234/ccdcw2021.222 PMC8563329

[32] Wang D, Liu A, Zhang S, Yu Y, Hu W, Sun X. History of the development of the reporting system of occupational diseases and occupational disease list in China. China CDC Wkly. 2020 May 1;2(18):314–8. 10.46234/ccdcw2020.080 34594646 PMC8422209

[33] Zhu X, Wang D, Wang H. Discussion on the status of statistical reporting and monitoring of occupational diseases and the construction of its information-based systems. Chin J Ind Med. 2018;31(1):73–5.

[34] [Inail. National mesothelioma registry (Renam)] [internet]. Rome: National Institute for Insurance against Accidents at Work; 2022. Italian. Available from: https://www.inail.it/cs/internet/attivita/ricerca-e-tecnologia/area-salute-sul-lavoro/sorveglianza-epidemiologica-negli-ambienti-di-lavoro-e-di-vita/renam.html?id1=6443101379561#anchor [cited 2022 Aug 15].

[35] [Inail. Malprof] [internet]. Rome: National Institute for Insurance against Accidents at Work; 2022. Italian. Available from: https://www.inail.it/cs/internet/attivita/ricerca-e-tecnologia/area-salute-sul-lavoro/sistemi-di-sorveglianza-e-supporto-al-servizio-sanitario-naziona/malprof.html?id1=6443099691915#anchor [cited 2022 Aug 15].

[36] [Inail. Injuries and occupational diseases claims and compensated cases] [internet]. Rome: National Institute for Insurance against Accidents at Work; 2022. Italian. Available from: https://www.inail.it/cs/internet/attivita/dati-e-statistiche/open-data.html [cited 2022 Aug 15].

[37] National occupational mortality surveillance South Africa (NOMS-SA) [internet]. Johannesburg: National Institute for Occupational Health; 2021. Available from: https://www.nioh.ac.za/national-occupational-mortality-surveillance-south-africa-noms-sa/ [cited 2022 Aug 24].

[38] NIDS [internet]. Cape Town: National Income Dynamics Study; 2020. Available from: http://www.nids.uct.ac.za/ [cited 2022 Aug 24].

[39] Mid-year population estimates 2021. Report No.: P0302. Pretoria: Statistics South Africa; 2021. Available from: https://www.statssa.gov.za/?page_id=1854&PPN=P0302&SCH=72983 [cited 2023 Apr 19].

[40] Statistics by theme, People: causes of death 2013 [internet]. Pretoria: Statistics South Africa; 2022. Available from: https://www.statssa.gov.za/?page_id=737&id=3 [cited 2023 Apr 19].

[41] The Births and Deaths Registration Act, 1992 (Act No. 51 of 1992) read with the Identification Act, 1997 (Act No. 68 of 1997). Available from: https://www.gov.za/documents/births-and-deaths-registration-act [cite 2023 Apr 24].

[42] Handbook on health inequality monitoring with a special focus on low- and middle-income countries. Geneva: World Health Organization; 2013. Available from: https://www.who.int/publications/i/item/9789241548632 [cited 2023 Apr 19].

[43] Health and environment country scorecards [internet]. Geneva: World Health Organization; 2022. Available from: https://www.who.int/teams/environment-climate-change-and-health/monitoring [cited 2022 Aug 11].

[44] EU strategic framework on health and safety at work 2012–2027. Occupational safety and health in a changing world of work. Brussels: European Commission; 2021. Available from: https://eur-lex.europa.eu/legal-content/EN/TXT/?uri=CELEX%3A52021DC0323&qid=1626089672913#PP1Contents [cited 2023 Apr 19].

[45] European occupational diseases statistics (EODS) [internet]. Luxembourg City: Eurostat; 2022. Available from: https://ec.europa.eu/eurostat/web/experimental-statistics/european-occupational-diseases-statistics [cited 2022 Aug 11].

[46] Hosseinpoor AR, Bergen N. Health inequality monitoring: A practical application of population health monitoring. In: Verschuuren M, van Oers H, editors. Population health monitoring: climbing the information pyramid. New York: Springer International Publishing; 2019. pp. 151–73. 10.1007/978-3-319-76562-4_8

[47] The effect of occupational exposure to solar ultraviolet radiation on malignant skin melanoma and nonmelanoma skin cancer: a systematic review and meta-analysis from the WHO/ILO joint estimates of the work-related burden of disease and injury. Geneva: World Health Organization; 2021. Available from: https://apps.who.int/iris/handle/10665/350569 [cited 2023 Apr 19].

[48] Loomis D, Dzhambov AM, Momen NC, Chartres N, Descatha A, Guha N, et al. The effect of occupational exposure to welding fumes on trachea, bronchus and lung cancer: a systematic review and meta-analysis from the WHO/ILO joint estimates of the work-related burden of disease and injury. Environ Int. 2022 Dec;170:107565. 10.1016/j.envint.2022.107565 36402034

[49] Pega F, Govindaraj S, Tran NT. Health service use and health outcomes among international migrant workers compared with non-migrant workers: A systematic review and meta-analysis. PLoS One. 2021 Jun 9;16(6):e0252651. 10.1371/journal.pone.0252651 34106987 PMC8189512

[50] Naicker N, Pega F, Rees D, Kgalamono S, Singh T. Health services use and health outcomes among informal economy workers compared with formal economy workers: a systematic review and meta-analysis. Int J Environ Res Public Health. 2021 Mar 19;18(6):3189. 10.3390/ijerph18063189 33808750 PMC8003536

[51] WHO Director-General’s keynote speech at the opening ceremony of the World Health Summit – 16 October 2022 [internet]. Geneva: World Health Organization; 2022. Available from: https://www.who.int/director-general/speeches/detail/who-director-general-s-keynote-speech-at-the-opening-ceremony-of-the-world-health-summit---16-october-2022 [cited 2023 Apr 19].

